# Deep Sequencing of Organ- and Stage-Specific microRNAs in the Evolutionarily Basal Insect *Blattella germanica* (L.) (Dictyoptera, Blattellidae)

**DOI:** 10.1371/journal.pone.0019350

**Published:** 2011-04-28

**Authors:** Alexandre S. Cristino, Erica D. Tanaka, Mercedes Rubio, Maria-Dolors Piulachs, Xavier Belles

**Affiliations:** 1 The Queensland Brain Institute, The University of Queensland, St. Lucia, Queensland, Australia; 2 Institute of Evolutionary Biology (CSIC-UPF), Passeig Maritim de la Barceloneta, Barcelona, Spain; East Carolina University, United States of America

## Abstract

**Background:**

microRNAs (miRNAs) have been reported as key regulators at post-transcriptional level in eukaryotic cells. In insects, most of the studies have focused in holometabolans while only recently two hemimetabolans (*Locusta migratoria* and *Acyrthosiphon pisum*) have had their miRNAs identified. Therefore, the study of the miRNAs of the evolutionarily basal hemimetabolan *Blattella germanica* may provide valuable insights on the structural and functional evolution of miRNAs.

**Methodology/Principal Findings:**

Small RNA libraries of the cockroach *B. germanica* were built from the whole body of the last instar nymph, and the adult ovaries. The high throughput Solexa sequencing resulted in approximately 11 and 8 million reads for the whole-body and ovaries, respectively. Bioinformatic analyses identified 38 known miRNAs as well as 11 known miRNA*s. We also found 70 miRNA candidates conserved in other insects and 170 candidates specific to *B. germanica*. The positive correlation between Solexa data and real-time quantitative PCR showed that number of reads can be used as a quantitative approach. Five novel miRNA precursors were identified and validated by PCR and sequencing. Known miRNAs and novel candidates were also validated by decreasing levels of their expression in dicer-1 RNAi knockdown individuals. The comparison of the two libraries indicates that whole-body nymph contain more known miRNAs than ovaries, whereas the adult ovaries are enriched with novel miRNA candidates.

**Conclusions/Significance:**

Our study has identified many known miRNAs and novel miRNA candidates in the basal hemimetabolan insect *B. germanica*, and most of the specific sequences were found in ovaries. Deep sequencing data reflect miRNA abundance and dicer-1 RNAi assay is shown to be a reliable method for validation of novel miRNAs.

## Introduction

In recent years, the discovery of short non-coding RNAs has changed the way molecular biologists understand the regulation of gene expression in almost all biological processes. The small RNA world include several kinds of short RNAs, such as microRNAs (miRNAs, ∼16–29 nt) [1] small interfering RNAs (siRNAs, ∼21 nt), and Piwi-associated RNAs (piRNAs, ∼24–30 nt), which regulate gene expression at the post-transcriptional level [2]. miRNAs play critical roles in many biological processes by repressing gene expression at the post-transcriptional level through binding usually at the 3′-untranslated region of the target mRNA [3], [4]. They comprise up to 5% of animal genes and may be involved in the regulation of one-third of the genes, thus being considered as one of the most abundant classes of small RNA regulators [5]. The other two classes of small RNAs (siRNAs and piRNAs) have only recently been identified in a number of metazoan species and may also play important roles in the regulation of gene expression [6], [7], [8]. All three classes of small RNAs are known to be implicated in RNA degradation pathways, thus disrupting the expression of endogenous genes (miRNAs and siRNAs), as well as in the control of transposable elements (siRNAs and piRNAs) [9], [10], [11].

Concerning insects, repertoires of small RNAs have been mainly established for species with their whole genome sequenced, such as 12 *Drosophila* species [12], three mosquitoes (*Anopheles gambiae*, *Aedes albopictus* and *Culex quinquefasciatus*) [13], [14], two hymenopterans (*Apis mellifera* and *Nasonia vitripennis*) [15], the flour beetle (*Tribolium castaneum*) [16], [17] and the silkworm (*Bombyx mori*) [18], [19]. They are all holometabolan insects and only recently, small RNAs have been identified using high throughput sequencing in the migratory locust (*Locusta migratoria*) [20] and in the pea aphid (*Acyrthosiphon pisum*) [21], which are hemimetabolan species.

The German cockroach, *Blattella germanica*, is one of the least-derived species in the evolution of insects and belongs to one of the most basal insect orders (Dictyoptera). It is being used as model of the hemimetabolan mode of metamorphosis [22], [23], in which growth and maturation occur simultaneously throughout successively nymphal stages until the imaginal molt, without an intermediate pupal stage. *B. germanica* is also a useful model to study the reproduction regulated by juvenile hormone, by which the batch of basal oocytes mature synchronously in each gonadotrophic cycle under the influence of circulating juvenile hormone levels [24], [25]. More recently, we have discovered that miRNAs play a key role in regulating metamorphosis [26] and oogenesis (E. D. Tanaka and M. D. Piulachs, unpublished) in *B. germanica*, and this lead us to gather more information of this class of small RNAs in our cockroach model. The present work reports the first results on the characterization of two small RNA libraries, one obtained from the whole body of the last instar nymph, which is the metamorphic stage, and the other one on adult ovaries, where oogenesis and vitellogenesis takes place. We followed the approach of high throughput sequencing using Solexa methodologies, and the results, beyond being useful for our functional research projects on metamorphosis and oogenesis, revealed surprisingly interesting data when comparing miRNA profiles in a specific tissue and in the whole body.

## Results and Discussion

### Construction of *B. germanica* small RNA libraries

**Figure 1:**
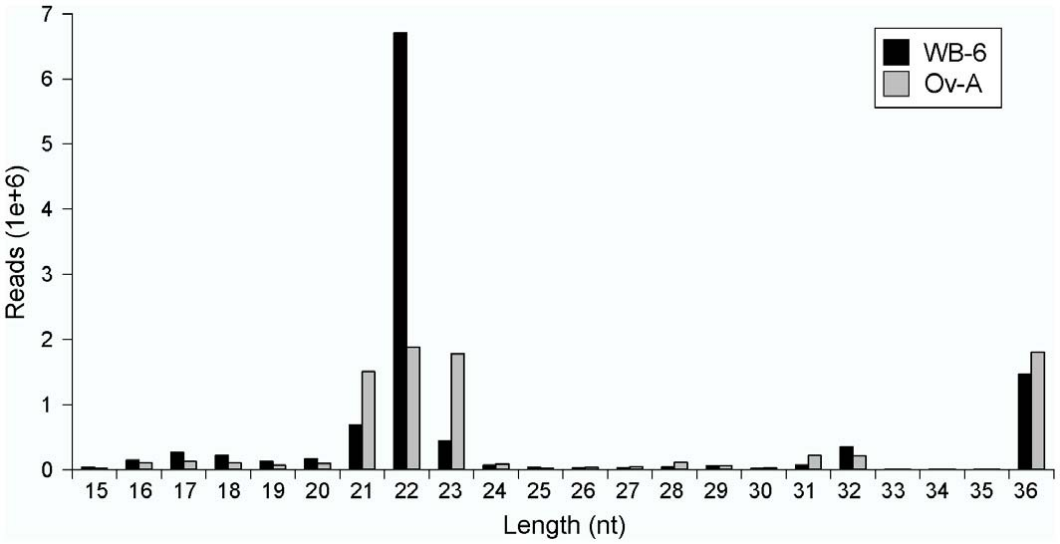
Length distribution of sequences. Length distribution of sequence reads obtained in the WB-6 and in the Ov-A libraries of *Blattella germanica*. Values are based on the raw data from Solexa sequencing.

We used Solexa sequencing technology to identify miRNAs in the cockroach *B. germanica*. Two libraries of small RNAs were constructed, one from the whole body (except head and digestive system) of sixth instar nymph females (WB-6) and the other one from adult ovaries in the first gonadotrophic cycle (Ov-A). The raw data are available at Gene Expression Omnibus (GSE22892). We obtained 10,824,998 reads (2,526,942 unique sequences) from the WB-6 library, and 8,190,720 reads (2,190,885 unique sequences) from the Ov-A. The sequence length distribution in WB-6 and Ov-A shows that both libraries are enriched with small RNAs of 21–23 nt (72% and 63% of all reads in WB-6 and Ov-A, respectively; Figure 1), which is considered the standard size of miRNAs. Another type of sequence found in both libraries was that of 36 nt-long RNAs, which represented 13 and 22% of the reads in WB-6 and Ov-A libraries, respectively. Of note, a peak at 22-nt size was also observed in small RNA libraries of *L. migratoria* [20], *A. albopictus* and *C. quinquefasciatus* [13]. In *B. mori*, small RNA libraries of feeding larvae, spinning larvae, pupae and adult [19], two peaks of length distribution were obtained, one around 20–22 nt, corresponding to miRNAs, and the other around 26–29 considered to contain pi-RNA-like sequences. Nevertheless, in the libraries of these four species, sequence size was limited to a maximum of 28–30 nt, therefore, we cannot ascertain whether they have a significant portion of 36-nt RNAs, as occurs in our small RNA libraries of *B. germanica*.

Low-quality sequences and those smaller than 19 nucleotides were removed from our data, thus remaining 9,161,578 reads (1,573,294 unique sequences) from WB-6 library, and 7,417,291 reads (1,682,864 unique sequences) from Ov-A. Only those sequences with more than 5 reads were used for further analyses as the distribution of reads per sequence shows that this number of reads is most close to the asymptotic line (Figure S1). Then, the sequences were clustered based on sequence similarity, taking into consideration that variations are often found at both 5′ and 3′ ends [13]. The variation of the 5′ end can be explained by imprecise or alternative processing by RNase III enzymes. The 3′ ends often contain untemplated nucleotide (most of them uracil and adenine), which must be added after processing by unknown terminal uridyl/adenyl transferases [2]. The filtering and clustering steps reduced the 36-nt RNAs to 5% and 7% of the reads in WB-6 and Ov-A libraries, respectively. The low abundance of 36-nt RNAs in the pre-processed data, where 99% of those sequences have only one read, indicates that they are most likely to be artefacts. In fact only a small proportion of reads (∼0.1%) partially matched to known sequences of ribosomal and other non-coding RNAs, bacterial genome and expressed sequence tags of *B. germanica* (Irles et al., 2009; X. Bellés and M. D. Piulachs, unpublished RNA libraries from *B. germanica*). Still the poor coverage of the alignments is due to a low-complexity tail on the 3′ end (the last 10 nucleotides) of those 36-nt RNAs which is mainly constituted by uracil (52%). Thus, only the sequence reads of miRNA length (21–23-nt) are of primary interest for the current study, and other small RNAs sizes are not considered in the following analysis.

### Identification of known miRNAs

**Figure 2:**
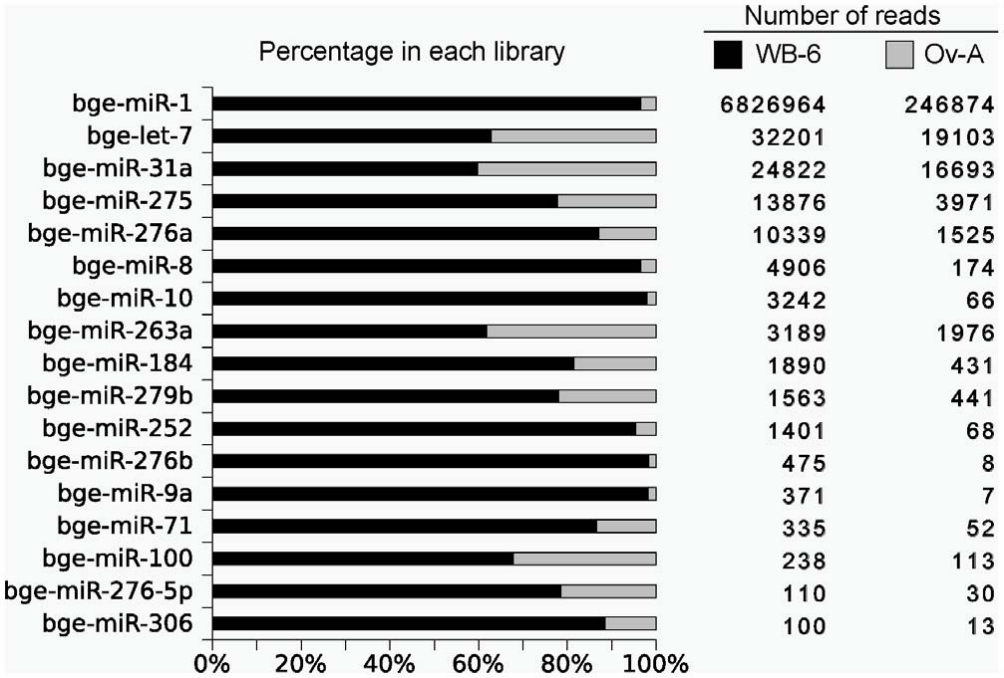
The most abundant known miRNAs identified in *Blattella germanica*. Known miRNAs with more than 100 reads found in the WB-6 and Ov-A *Blattella germanica* libraries.

Firstly, our *B. germanica* libraries were examined in search for miRNAs which had been recognised in other species. A total of 38 known miRNAs were identified in the WB-6 and Ov-A libraries (Table S1). Figure 2 summarizes the known miRNAs with more than 100 reads detected in our libraries. The most abundant was miR-1, with *ca.* 7 million reads. It was followed by let-7, miR-31a, miR-275 and miR-276a, with a number of reads comprised between 10,000 and 30,000. However, the distribution of these reads amongst the two libraries, WB-6 and Ov-A, was different as we will report in detail later. Some of the known miRNAs have been found as the most abundant ones in other insect species, such as miR-1, miR-275, miR-276 and miR-8 in *L. migratoria* [20]; and miR-1, miR-8, miR-276a and miR-263a in *B. mori* [19]. In the mosquitoes *A. albopictus* and *C. quinquefasciatus*, the most highly expressed miRNA is miR-184, and these two species shared three out of ten most abundant miRNAs in *B. germanica* libraries: miR-184, miR-275, and miR-8 [13].

Concerning the function of the more abundant miRNAs found in our libraries, miR-1 is one of the most conserved miRNA across metazoans, and is continuously expressed during the embryogenesis and development of *Drosophila melanogaster* and *B. mori* [27], [28]. The best characterised functions of miR-1 are related to the regulation of genes involved in muscle function [29]; in *D. melanogaster* it is required for post-mitotic growth of larval muscle [30], targeting mRNAs that impair muscle development [28]. Let-7 has been one of the most studied miRNAs since its discovery in the nematode *Caenorhabditis elegans*. The expression pattern, which is conserved in many species, suggests that let-7 might control temporal transitions during development [31]. Let-7 clusters with miR-100 and miR-125 in the same primary transcript in most insects species [31], [32]. In *D. melanogaster*, the expression patterns of let-7 and miR-125 correlate with titer profiles of 20-hydroxyecdysone and juvenile hormone and the end of larval life, which suggest that these miRNAs are involved in the regulation of metamorphosis [33], [34], [35]. Similar observations have been made in *B. mori*, although in this lepidopteran, let-7 is stage- and tissue-specifically expressed, and it appears from the first molt, which suggests that it might play regulatory roles at early larval stages. The detailed expression profiles in the whole life cycle and in cultured cell lines of silkworm showed a clear association with ecdysone pulses and with a variety of biological processes [36], [37]. In *D. melanogaster*, it has been shown that let-7 targets the transcripts of the *abrupt* gene, thus preventing its expression in the muscle and allowing a correct maturation during metamorphosis [38]. Indeed, one of the functions of the complex let-7, miR-100 and miR-125 is to ensure the appropriate remodelling of the abdominal musculature during the larval to adult transition [39]. To date, the majority of miRNA expression data comes from studies with the silkworm *B. mori*. For example miR-31a shows continuous high expression from the spinning larval to pupal and adult stages. Also, miR-275 is up-regulated from the beginning of the third instar to pupa and down-regulated during pupal metamorphosis of both sexes, while miR-276 which is one of the most abundant miRNAs in *B. mori*, is preferentially expressed in feeding and spinning larvae [19], [27].

### Identification of known miRNA*

We also searched for conserved complementary sequences of mature miRNA, known as miRNA stars (miRNA*s). A total of 11 conserved miRNA*s were identified based on homologous precursor miRNAs from different insect species (Table S2). miR8* was the most abundant (*ca.* 50,000 reads), followed by miR-276a* (*ca.* 10,000 reads) and by miR-10* (*ca.* 1000 reads). Interestingly, some of these miRNA* (like miR-8*, 276-5p* and miR-993a*) have roughly the same or even higher number of reads than their respective mature miRNA. Of note, miR-965* (357 reads), miR-9b* (43 reads), miR-281* (1444 reads) and miR-9c* (138 reads) were identified in our libraries, whereas their respective mature miRNA counterparts were not found.

Various miRNA* sequences have been detected in high amounts in other libraries of small RNA [19], [20], [40]. Moreover, a number of miRNA*s detected in the *B. germanica* libraries are also present in those of *L. migratoria* (miR-281*, miR-10*, miR-8*) [20] and *B. mori* (miR-10*, miR-281*) [19], [41]. Arguably, the high number of miRNA* reads and the degree of conservation suggest an evolutionary constraint, which in turn indicates they must play some function [12], [42]. In *D. melanogaster*, for example, miR-10* is more abundant than miR-10, and both have conserved target sites in Hox genes [12], which suggests that they interplay to regulate developmental processes.

### Quantitative value of Solexa sequencing data

The number of reads resulting from deep sequencing is often considered a reliable quantification of miRNA expression [19]. However, this is a hypothesis *a priori* which has not been empirically tested yet. In order to evaluate whether our Solexa sequencing data have a quantitative value, we carried out qPCR analysis of known miRNAs (15 for the WB-6 library and 12 for the Ov-A), using the same RNA samples used to construct the libraries. The selection criterion for known miRNAs was to cover a wide spectrum of reads number.

**Figure 3:**
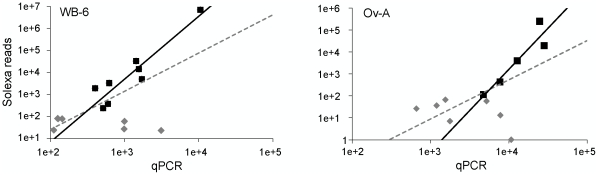
Quantitative value of Solexa reads. The number of reads of Solexa sequencing and fold change of qPCR analysis (miRNA copies per 1000 copies of U6) are correlated. The two *Blattella germanica* miRNA libraries, WB-6 and Ov-A are indicated. Squares are known miRNA with more than 100 Solexa reads. Diamonds are miRNAs with less than 100 reads. Solid lines are trend line for correlation with 100-more Solexa reads. Dashed lines are trend line for all miRNAs tested.

We used Pearson's correlation to test if the numbers of Solexa reads and qPCR experiments (Figure 3 and Table S3) were correlated. The results indicate that when all miRNAs were considered into the analysis, there was no significant correlation between Solexa reads and qPCR data. However, a significant positive correlation was observed when we considered those miRNAs with more than 100 Solexa reads in both WB-6 (R = 0.93, P-value<0.01) and Ov-A libraries (R = 0.93, P-value<0.05) (Figure 3). These results suggest that Solexa sequencing data with less than 100 reads can only roughly represent the relative abundance of miRNAs, therefore we used only sequences over 100 reads for further comparisons between WB-6 and Ov-A libraries.

### Identification of novel miRNA candidates

Many other small RNA sequences were found highly represented in the WB-6 and Ov-A libraries and arguably they are likely to be functional small RNAs. A total of 1,428 different sequences with at least 100 reads were identified in our Solexa data (Table S4). As most of the 1,428 sequences were very similar to each other we followed a clustering approach to reduce redundancy. Thus, we used the transitivity clustering method [43] to create a graph that depicts the pairwise similarity between each candidate by representing the sequences as nodes and the similarity as links (Figure S2). We also used complex network methods to construct a non-redundant database of miRNA candidates by selecting the most abundant sequences as representative of each cluster. The whole similarity network is divided in 224 components, being one major component composed by 1099 sequences (Figure S2). The major network shows virtually all possible variants for the most expressed candidate (bge_candidate 1 = 4,585,181 reads); however, the modular structure of the network shows that some candidates are clustered in sub-structures (modules), which indicate that they might be real sequences rather than spurious variations of bge_candidate 1. We objectively measured the modularity of the large network (modularity = 0.74) and found a total of 15 modules (see Text S1 for modules composition), which were considered as different clusters; therefore, the 1099 redundant sequences were reduced to 15 candidate sequences. Other 41 obvious clusters have also been represented by their most frequent reads. The clustering analysis finally resulted in a total of 240 non-redundant candidate sequences, which were used for further analysis (Table S5). These candidate sequences are among the most highly expressed small RNAs in *B. germanica* and are likely to have some functional role. They are small sequences of 21 to 23 nt in length where 70% is 22 nt-long. About a third (70) of those sequences were also found in other insects that had been subjected to high throughput sequencing for small RNAs, such as *L. migratoria* [20], *A. pisum* [21], *B. mori* [19], [40] and *C. quinquefasciatus* [13] (Table S5); these are herein called “conserved” candidates. However, most of those abundant small RNA sequences are, by the moment, unique of *B. germanica* (170 sequences), and have been called “specific” candidates (Table S5).

**Figure 4:**
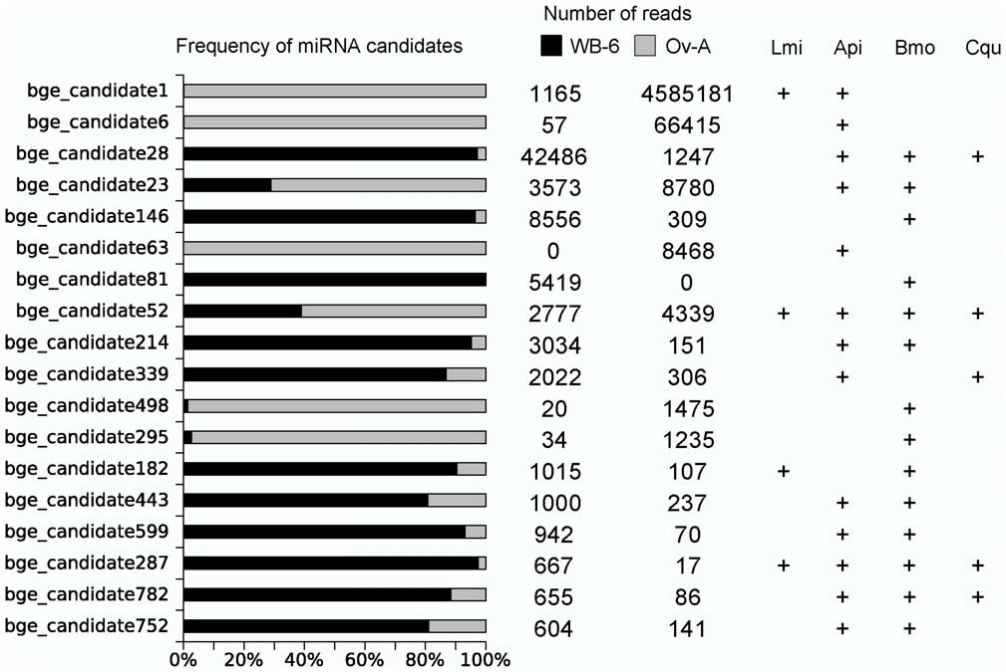
miRNA candidates conserved in other insect species. Conserved miRNA candidates found in the WB-6 and Ov-A *Blattella germanica* libraries with more than 500 reads. They have been reported in small RNA databases from other insects such as *Locusta migratoria* (Lmi), *Bombyx mori* (Bmo), *Acyrthosiphon pisum* (Api) and *Culex quinquefasciatus* (Cqu) (see also Table S5).

Figure 4 shows the frequency of the most abundant (at least 500 reads) conserved candidates in the WB-6 and Ov-A libraries, and their presence or absence in the aforementioned insect species. Highly similar small RNA sequences were found in *A. pisum* (48 sequences) [21], *B. mori* (34 sequences) [40], *C. quinquefasciatus* (9 sequences) [13] and *L. migratoria* (11 sequences) [20] (Table S5). In these data, co-occurrence of small RNAs do not reflect evolutionary relationships, as it is known that *B. germanica* is phylogenetically closer to *L. migratoria* than to *A. pisum* or *B. mori*. Rather, differences in presence/absence of small RNA sequences derive from differences in the experimental approaches by sampling different tissues and stages as well as the “deepness” of the sequencing methods.

Deep sequencing projects led to discover a higher number of unique sequences, as in the case of *B. germanica* libraries, which accounted for *ca.* 18 million reads giving 240 miRNA candidates. The *A. pisum* library, with about 0.9 million reads, gave 94 novel miRNA candidates [21]. *B. mori* is one of the best studied species in this sense, with successive stage- and organ-specific small RNA libraries reported [40], [41], with the last one accounting for *ca.* 4 million reads and leading to the discover of 287 novel miRNA candidates [41]. Two *L. migratoria* libraries, one for the solitary phase and another for the gregarious phase, accounted for *ca.* 3.5 million reads and gave 185 novel miRNA family candidates [20].

### Identification of novel miRNA precursors and validation by PCR amplification

**Figure 5:**
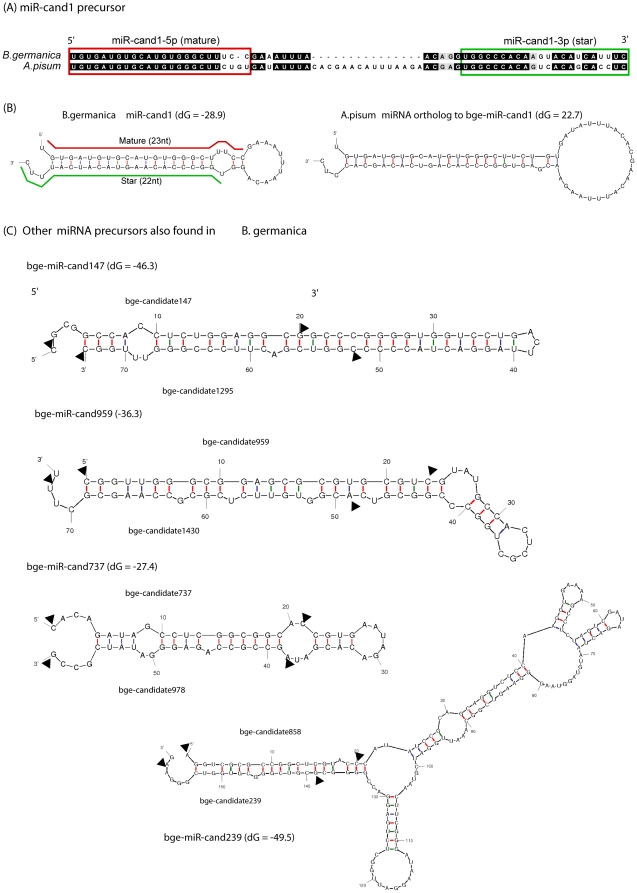
miRNA precursors validated by PCR amplification and sequencing. (A) The most abundant miRNA, bge-miR-cand1, in the cockroach ovaries is also conserved in the aphid *A. pisum*. (B) Secondary structure of miR-cand1 hairpin in both cockroach and aphid. (C) Other four miRNA precursors showing the location of novel miRNAs in *B. germanica*.

As the genome of *B. germanica* has not been sequenced yet, the identification and validation of miRNA precursors becomes an interesting challenge. We used two approaches to validate the precursors of some miRNA candidates found highly abundant in our two small RNA libraries, WB-6 and Ov-A. In the case of the bge-miR-cand1, the precursor sequence was obtained after aligning with *A. pisum* genome sequences (Figure 5A), designing appropriate primers (Table 1) and amplifying the precursor by PCR, using RNA extracts from adult ovaries as template. The precursor sequence of the most abundant miRNA candidate in adult ovaries, bge-candidate 1, was characterized by this method (Figure 5A–B) and only a few copies of its miRNA* could be found in Ov-A library (see Text S2). Interestingly bge-miR-cand1 was also found highly conserved in the aphid *A. pisum* genome [44] which is a hemimetabolan with panoistic ovaries, just as *B. germanica*.

**Table 1:** Validated *Blattella germanica* miRNA precursors.

miRNA-5prime		
miRNA name	ID	Sequence
bge-miR-cand1-5p	bge_candidate1	5′TGTGATGTGCATGTGGGCTTTCC3′
bge-miR-cand147-5p	bge_candidate147	5′CGCGGCCACCTCTGGAGGCGG3′
bge-miR-cand239-5p (*)	bge_candidate858	5′AGAGGTCGCGCCGGCTCGTACC3′
bge-miR-cand737-5p	bge_candidate737	5′CACAGATAGCCTCGGCGGCACC3′
bge-miR-cand959-5p	bge_candidate959	5′CGGTTGGGCGGAGCGCGTGCGTC3′

miRNA-3prime		
bge-miR-cand1-3p (*)	bge_candidate1429	5′TGGCCCACAAGTACATCATTTC3′
bge-miR-cand147-3p (*)	bge_candidate1295	5′CGGTCGACTTCCCGGGTTTGGC3′
bge-miR-cand239-3p	bge_candidate239	5′CGCGTCGGGCGTGGTCGGGAAGC3′
bge-miR-cand737-3p (*)	bge_candidate978	5′AGCCGCCAGAGGGATATCGCCG3′
bge-miR-cand959-3p (*)	bge_candidate1430	5′ACGGTGGTCTCGCGCCAAGCGCTT3′

(*)indicates the putative star based on frequency of reads.

Previous studies have reported that full-length of small non-coding RNAs can be often reconstructed through assembling short reads for identification of novel non-coding RNA candidates [45], [46]. Therefore, we used a similar approach by aligning all 240 miRNA candidates against a database of RNA sequences from *B. germanica* constructed by integrating the two RNA libraries of the present study (WB-6 and Ov-A), and other five libraries of small RNA sequences from whole-body and ovaries of cockroaches at different developmental stages and water deprivation treatments (X. Bellés and M. D. Piulachs, unpublished RNA libraries from *B. germanica*) (Figure S3). All candidate sequences were aligned against this integrated *B. germanica* RNA database in order to identify potential miRNA precursors among those contigs. Only those contigs with at least two sequences located in different regions were further checked for folding stability using mfold [47]. The potential miRNA:miRNA* duplexes were then predicted by looking for candidate sequences located in opposite arms of hairpins (Table 1). Four novel miRNA precursors have been found among the contigs in the *B. germanica* RNA database (Figure 5C; see also Figure S3).

These five miRNA precursors were validated by PCR amplification and sequencing which confirmed the putative miRNA precursors (Figure 5). All sequence reads from WB-6 and Ov-A libraries were mapped into the contigs and the distribution of those reads across the contigs indicates that they are not explained by the degradation process as the pattern of expression of those small RNAs found in our libraries vary in whole body and ovaries (Text S2). That indicates they are differentially processed in different developmental stages and tissues.

Interestingly we could not found any known miRNAs in *B. germanica* RNA database as well as bge-candidate 1 precursor, which indicates that some canonical miRNAs have a more precise processing than other miRNAs by producing mostly mature miRNA sequences and some miRNA*s but barely a sequence from the loop region.

The two approaches, alignments and PCR, and de novo assembly, used in our study seems to be reliable methods for the experimental validation of the novel miRNA precursors. In this manner it is possible to identify and characterize novel miRNAs on those species without genome sequenced.

### Validation of miRNA candidates by dicer-1 RNAi

Dicer ribonucleases are important in the biogenesis of miRNAs as they are involved in the production of mature miRNAs from precursor miRNAs (pre-miRNAs), and of small interfering RNAs (siRNAs) in the RNA interference (RNAi) pathway [48]. A single dicer ribonuclease is involved in both miRNA and siRNA production in the nematode *C. elegans* and vertebrates. However, two dicer ribonucleases, dicer-1 and dicer-2, exist in insects and act in the miRNA and siRNA pathways, respectively [48]. Previous studies have shown that dicer-1 RNAi in *B. germanica* specifically decreases the expression levels of mature miRNAs [26]. Therefore, dicer-1 silencing might be a useful approach to validate miRNA candidates in those species with no sequenced genome, given that in principle only those small RNAs whose levels became lowered after dicer-1 RNAi would be miRNAs.

**Figure 6:**
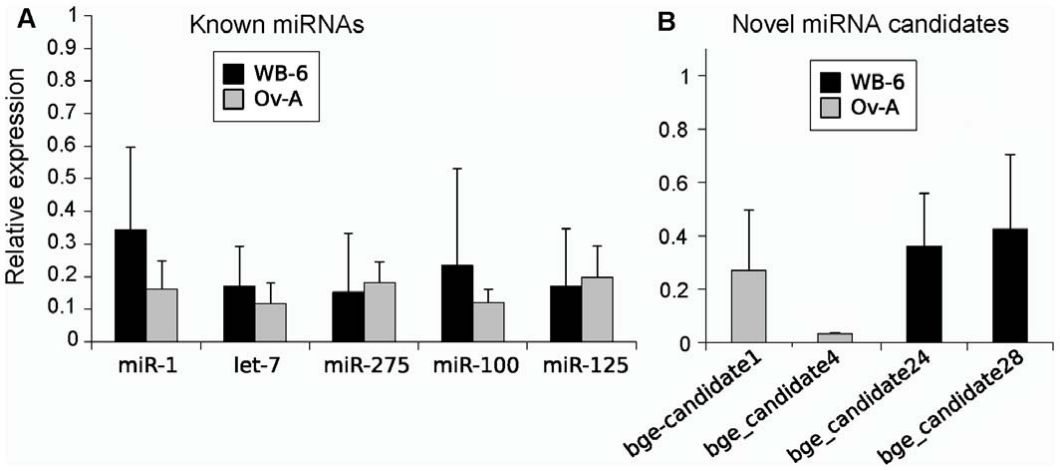
Effect of dicer-1 knockdown on miRNA expression. Effect of dicer-1 knockdown in the expression of known miRNAs (A) and miRNA candidates (B) of *Blattella germanica*. Data showed are the values obtained in dicer-1 knockdown animals normalized against the control (reference value = 1). Differences with respect to control were statistically significant in all cases (p<0.05), according to the REST© software tool (see the Table S6). The error-bars show standard deviation for three biological replicates.

Using dicer-1 RNAi, we first measured the expression levels in a representation of known miRNAs (miR-1, let-7, miR-100, miR-125 and miR-275) in sixth nymphal instar whole bodies and in adult ovaries from individuals treated with dsRNA targeting dicer-1. Figure 6A shows the significant decrease of miRNA levels in both groups. Then we used the same dicer-1 RNAi assay to test 4 novel miRNA candidates, being 2 of them conserved (bge_candidate-1, and bge_candidate-28) and 2 specific (bge_candidate-4 and bge_candidate 24-). These experiments suggest that these 4 novel miRNA candidates are real miRNAs (Figure 6B, Table S6). We used the gene 5S as negative control as its expression was unaltered after dicer-1 silencing (results not shown).

### Other small RNA sequences

We also found other small RNAs in our libraries such as piRNA-like sequences and transposon fragments as well as fragments of genomic DNA of *Blattabacterium* sp., a bacterial endosymbiont of *B. germanica* [49]. However, these sequences were found in small number in the cockroach libraries, representing 0.7% of the WB-6 (0.4% piRNA/transposons and 0.3% bacterial) and 0.4% of the Ov-A libraries (0.2% piRNA/transposons and 0.2% bacterial).

Of particular interest are those sequences derived from the *Blattabacterium* sp. genomic DNA. This bacterium is an intracellular endosymbiont of cockroaches and primitive termites which plays central role in enhancing the metabolic capacity of their hosts to live on nutrient-deficient diets [50]. The bacterial sequences found in our libraries (Table S7) correspond to regions regularly distributed across the *Blattabacterium* circular genome (Figure S4). The most plausible hypothesis is that these sequences are products of degradation, although we cannot discard the possibility that some of them, especially those that have a higher number of reads, might play some function over the bacteria or their host. These findings indicate again that different types of small RNAs can be identified by Solexa deep sequencing, which shows that many other processes regulated by small RNAs are still waiting for further characterization.

### Comparison of the two cockroach libraries

**Figure 7:**
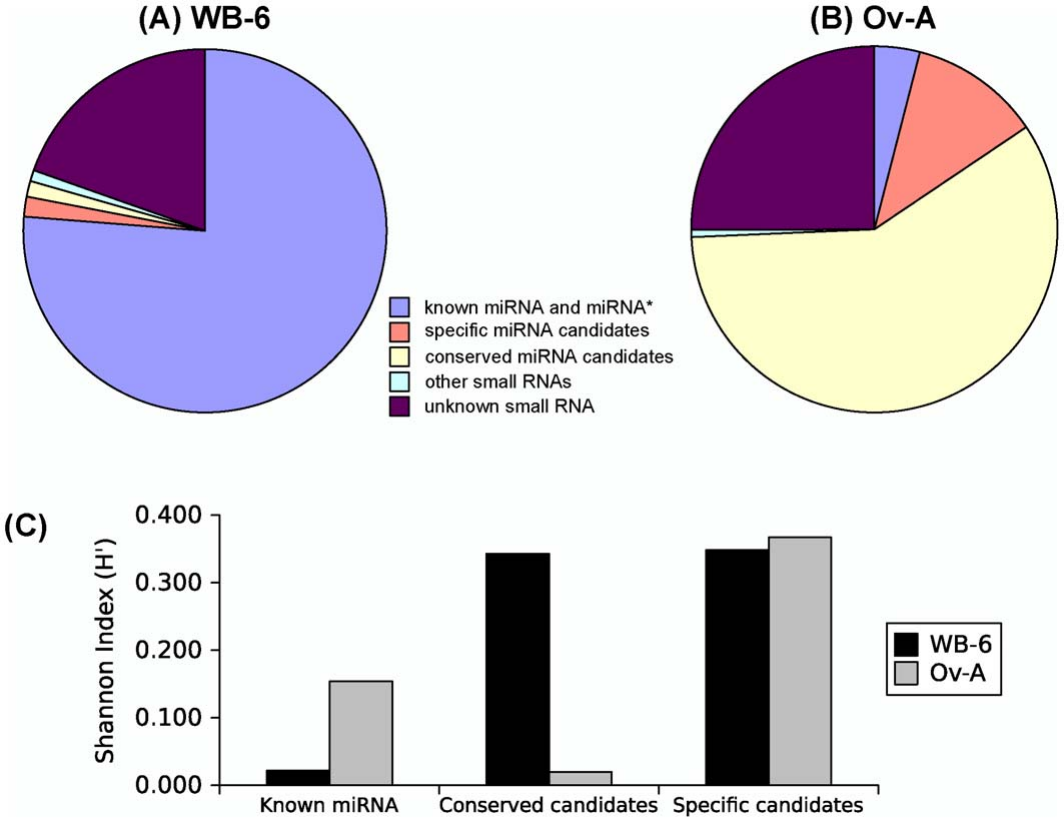
Comparison of WB-6 (A) and Ov-A (B) libraries. A–B: Distribution of small RNAs in the WB-6 (A) and Ov-A (B) *Blattella germanica* libraries. “Other small RNAs” states for rRNAs, tRNAs, ncRNAs, piRNAs-like and *Blattabacterium* sp. sequences. (C) The Shannon diversity index (H′) estimated from WB-6 (A) and Ov-A (B) libraries considering the known miRNAs, conserved candidates and specific candidates.

The comparison of the two libraries, WB-6 and Ov-A, provides also important insights regarding the small RNA composition in the whole body and in a single tissue. Concerning the known miRNAs, the WB-6 library has a much higher number of reads (6,926,591 reads) than the Ov-A (291,829 reads). Moreover, the known miRNAs are clearly over-represented in the WB-6 library. More than 95% of the reads of miR-1, miR-8, miR-252, miR-276b, and miR-9a were obtained from the WB-6 library, and the catalogue of known miRNAs is richer in the WB-6 library as miR-317, miR-993a, miR-34, miR-14, miR-315, miR-iab-4, miR-375, miR-190 and miR-iab-4as-5p are present in this library but not in Ov-A. Conversely, only miR-12 is present in the Ov-A library but not in the WB-6 (Figures 2, 7A, B).

In contrast, the number of reads of miRNA candidates (conserved and specific) is much higher in the Ov-A (5,215,516 reads) than in the WB-6 library (290,763 reads). The candidate miRNAs are over-represented in the Ov-A library, and only few candidates have a substantial number of reads in the WB-6 library (Figures 4, 7A, B). Another important difference between these two libraries concerns the diversity of miRNAs. We have calculated the classical diversity index of Shannon based on the proportion of number of reads per sequence for known miRNA and conserved and specific candidates. We found that Ov-A library is more diverse than WB-6 in terms of known miRNAs. However the diversity in Ov-A library is lower than in WB-6 for the conserved novel miRNA candidates, while both libraries show similar diversity for specific candidates (Figure 7C).

In fact, WB-6 library is enriched with known miRNAs, while Ov-A is preferentially enriched with novel miRNA candidates, mainly specific ones thus suggesting that ovarian development in the adult might require particular miRNAs that there are not expressed in the whole body of the last instar nymph. However, nymphal ovaries are also represented in the WB-6 library, which suggests (although nymphal and adult ovaries are not functionally equivalent) a dilution effect in the WB-6 library. Thus, those miRNAs feebly represented in the whole body extract would be missing by the deep sequencing. This pinpoints the relevance of making organ-specific libraries if the aim is to work with a particular organ and to get robust conclusions at miRNA level. A corollary of the hypothesis of the dilution effect is that the deeper the sequencing the better the coverage, given that most feebly represented miRNAs will be revealed by the analysis. Thus, our approach of deep sequencing of tissue versus whole body samples (*ca.* 18 million reads) has been crucial to reveal a high number of small RNA sequences that have not been described in any previous study. The way is therefore paved for undertaking the task of studying the functional sense of such huge miRNA diversity in our singular cockroach model.

## Materials and Methods

### Animals, samples preparation and sequencing approach

Freshly ecdysed sixth (last) instar nymphs and adult females of the cockroach *B. germanica* were obtained from a colony reared in the dark at 30±1°C and 60–70% relative humidity. For the WB-6 samples, the entire animal except the head (to avoid interferences with the eye pigments) and the digestive tube (to avoid contamination with parasites) was used. Stage specific samples of 2–3 individuals were collected for each of the 9 days of the last instar nymph. Then a pool composed by day 0 to 8 aliquots was built in order to cover the entire last instar nymph. For the Ov-A samples, we dissected the ovary pair from adult virgin cockroaches in each day of the first gonadotrophic cycle, which lasts 8 days. The pooling procedure to get an extract covering in this case the whole first gonadotrophic cycle was equivalent to that followed in the WB-6 extract. All dissections and tissue sampling were carried out on carbon dioxide-anaesthetized individuals. RNA isolation from WB-6 and Ov-A samples was carried out with mirVana miRNA Isolated kit (Ambion), which increases the yield of small RNAs. The total amount of RNA in WB-6 and Ov-A samples was approximately 10 µg. Sequencing was performed on an Illumina Genome Analyzer with Solexa technology.

### Analyses of Solexa data

We filtered the raw Solexa sequencing data to discard low quality sequences containing ambiguous nucleotides, as well as sequences smaller than 19 nucleotides. All the remaining sequences were clustered based on sequence similarity using BLASTCLUST program with 90% identity and 80% coverage. The sequence of the predominant read of each cluster was used for the following analyses. The non-redundant sequences were then aligned by BLASTN program against Solexa adaptors and primers as well as databases of tRNA [51], rRNA [52] and snRNA (FlyBase and GenBank).

Then, we searched for conserved miRNAs as described in miRBase [53]. All insect miRNAs in miRBase with one or more sequences were used to build PSSM profiles for each miRNA gene. A total of 210 profiles were constructed and used to search for miRNAs with at least 90% identity in the *B. germanica* small RNA libraries. The Solexa sequencing data has been described as a rich source of data for many small RNAs other than only miRNAs, such as mature miRNA complementary sequences (miRNA*), Piwi-like RNAs and small interference RNAs. We looked for conserved miRNA* in both libraries using BLASTN program to align all Solexa sequences against the hairpin sequences with mature miRNA region masked.

We also checked our libraries for novel miRNA candidates based on the abundance of reads and their sizes. To create a graph depicting the pairwise similarity between each candidate by representing the sequences as nodes and the similarity as links, we used transitivity clustering method [43]. The graph is created based on alignments were performed by BLASTN (90% identify and 95% coverage; e-value threshold <1e-05) and visualized as networks using Cytoscape [54] (Figure S2). Complex network analysis was conducted to make a non-redundant database of putative miRNA candidates by selecting the most abundant read as representative of each cluster. The network analysis was performed by igraph (http://igraph.sourceforge.net) and Python programming language (http://www.python.org). We used fast greedy modularity optimization algorithm for finding community (or modular) structure in the networks [55], [56] (Text S1).

In order to provide evidences for the novel miRNA candidates we aligned (BLASTN with 95% coverage and 100% identity) all *B. germanica* sequences against others insect small RNAs databases obtained by deep sequencing such as *L. migratoria* [20], *A. pisum* [21], *B. mori* [19], [40], *C. quinquefasciatus* and *A. albopictus* [13]. We also looked for all putative miRNA candidates in a database of assembled contigs of *B. germanica* constructed by the integration of the two small RNA libraries approached in the current study (WB-6 and Ov-A), and other five RNA libraries of whole-body and ovary from cockroaches in different developmental stages (X. Bellés and M. D. Piulachs, unpublished RNA libraries from *B. germanica*; Figure S3).

The assembly was performed by constructing a pipeline which uses different software for the assembly, Velvet [57], SOAPdenovo [58] and phrap (http://www.phrap.org) (Figure S3). To map the reads from the two libraries, WB-6 and Ov-A, into assembled contigs we used bwa [59] and SAMtools [60], and visualization of the mapping was done by Integrative genomics viewer [61] (Text S2).

For the identification of piRNA-like RNAs, all non-annotated sequences from *B. germanica* libraries were aligned against piRNA sequences found in the GenBank and *D. melanogaster* transposon database (FlyBase). To identify sequences from the bacterial endosymbiont *Blattabacterium* sp. [49] we aligned *B. germanica* small RNA sequences against *Blattabacterium* sp. genome (BLASTN with 95% coverage and 100% identity).

### Validation of predicted novel miRNA precursors by PCR amplification

After the assembly of contigs of *B. germanica*, novel miRNA precursors were identified and four of them were selected for cloning (bge-miR-cand147, bge-miR-cand239, bge-miR-cand737 and bge-miR-cand959). Primers were designed according to the sequences of miRNA showed in Table 1. Amplifications by PCR were carried out using as template cDNA from 4-day-old of sixth nymphal instar, which had been treated in fifth nymphal instar with dsRNA targeting dicer-1 (see the next section). Regarding to the bge-miR-cand1, the precursor sequence was obtained after alignment with *A. pisum* genome sequences and PCR amplifications were carried out using as template cDNA of ovaries from 3-day-old female adults which had been treated with dsRNA targeting dicer-1 just after the imaginal emergence. For all of those miRNA precursors, PCR conditions were as follows: 94°C for 3 min, followed by 40 cycles of 94°C for 30 s, 60°C for 30 s, 72°C for 30 s, and a final extension step at 72°C for 7 min. The amplification products were analyzed by electrophoresis in 2% agarose gels and the fragments with expected size were ligated into pSTBlue™-1 AccepTor™ Vector (Novagen®) for transformation of competent cells (pSTBlue™-1 AccepTor™ Vector Kit, Novagen®). DNA sequencing was performed by the dideoxy sequencing method, using a BigDye terminator v3.0 Cycle Sequencing Ready Reaction (Applied Biosystems) in an ABI Prism 310 Genetic Analyzer (Applied Biosystems).

### RNAi of dicer-1

To silence dicer-1 expression in *B. germanica* by RNAi, we prepared a dsRNA encompassing a 343 bp region placed between the RNAseI and RNAseII domains of BgDcr1 [26]. The dsRNA was injected at a dose of 3 µg in *B. germanica* females at the freshly emerged fifth (penultimate) nymphal instar or at the freshly emerged sixth (last) nymphal instar. As control dsRNA, we used a non-coding sequence from the pSTBlue-1 vector (dsMock) injected at a dose of 3 µg [26]. In the group treated on the penultimate nymphal instar, expression of dicer-1 was examined on whole body extracts on day 4 of the last nymphal instar, whereas in the group treated in last nymphal instar it was examined on the ovary of freshly emerged adults. In both cases, dicer-1 transcript levels significantly decreased *ca.* 60%, as in the experiments previously reported [26]. Three biological replicates were carried out of each experiment.

### Quantification of miRNAs by real time PCR

For mRNA expression studies by qRT-PCR, 400 ng of total RNA from the pools prepared for libraries were reverse transcribed using the NCode™ First-Strand cDNA Synthesis Kit (Invitrogen) following the manufacturer's protocols. Amplification reactions were carried out using IQ™ SYBR Green Supermix (BioRad) and the following protocol: 95°C for 2 min, and 40 cycles of 95°C for 15 s and 60°C for 30 s, in a MyIQ Real-Time PCR Detection System (BioRad). After the amplification phase, a dissociation curve was carried out to ensure that there was only one product amplified. All reactions were run in triplicate. Statistical analysis of relative expression results was carried out with the REST© software tool [62] (see the next section). Results are given as copies of RNA per 1000 copies of U6. Primer sequences are available on request.

### Statistics

The correlation between numbers of Solexa reads and PCR quantification was calculated by Pearson's correlation analysis and the statistical significance was assigned by Student's t test. Statistical analysis of expression values of particular miRNAs and candidates was carried out using the REST 2008 program (Relative Expression Software Tool V 2.0.7; Corbett Research) [62]. This program calculates changes in gene expression between two groups, control and sample, using the corresponding distributions of *Ct* values as input [62]. The program makes no assumptions about the distributions, evaluating the significance of the derived results by Pair-Wise Fixed Reallocation Randomization Test_ tool in REST [62].

## Supporting Information

Figure S1 **Distribution of reads per sequence for WB-6 and Ov-A libraries.**
(TIF)

Figure S2 **Graph showing pairwise similarity between all candidate sequences of** ***Blattella germanica*** **(see Table S4).** Nodes in orange are candidate sequences chosen to represent the cluster based on the highest amount of reads.
(EPS)

Figure S3 **Pipeline used for assembling the sequences using Velvet, SOAPdenovo and phrap softwares.**
(TIF)

Figure S4 **Distribution of the** ***Blattabacterium*** **sequences obtained in the libraries (highlighted in purple) across the** ***Blattabacterium*** **sp. genome (highlighted in turquoise blue).**
(TIF)

Table S1 Sequences and number of reads for known miRNA found in WB-6 and Ov-A libraries of *Blattella germanica*.
(XLS)

Table S2 Sequences and number of reads of known miRNA* found in WB-6 and Ov-A libraries of *Blattella germanica*.
(XLS)

Table S3 Comparison of miRNA abundance data obtained by qPCR and number of Solexa reads for a selection of known miRNAs.
(XLS)

Table S4 Complete list of miRNA candidates found in the WB-6 and Ov-A libraries of *Blattella germanica* before clustering analysis.
(XLS)

Table S5 Non-redudant miRNA candidates found in the WB-6 and Ov-A libraries of *Blattella germanica*.
(XLS)

Table S6 Statistical analysis of the experiments of dicer-1 RNAi to validate miRNA candidates.
(XLS)

Table S7 Small RNA sequences derived from *Blattabacterium* sp. genome.
(XLS)

Text S1 Clustering analysis of redundant miRNA candidates.
(DOC)

Text S2 Identification of miRNA precursors in *B. germanica* RNA databases.
(DOC)
