# Supplementary figures and images for: Deep Sequencing of Organ- and Stage-Specific microRNAs in the Evolutionarily Basal Insect Blattella germanica (L.) (Dictyoptera, Blattellidae)
Source: PLoS One. 2011 Apr 28;6(4):e19350. doi: 10.1371/journal.pone.0019350 (PMC3084283; doi:10.1371/journal.pone.0019350)

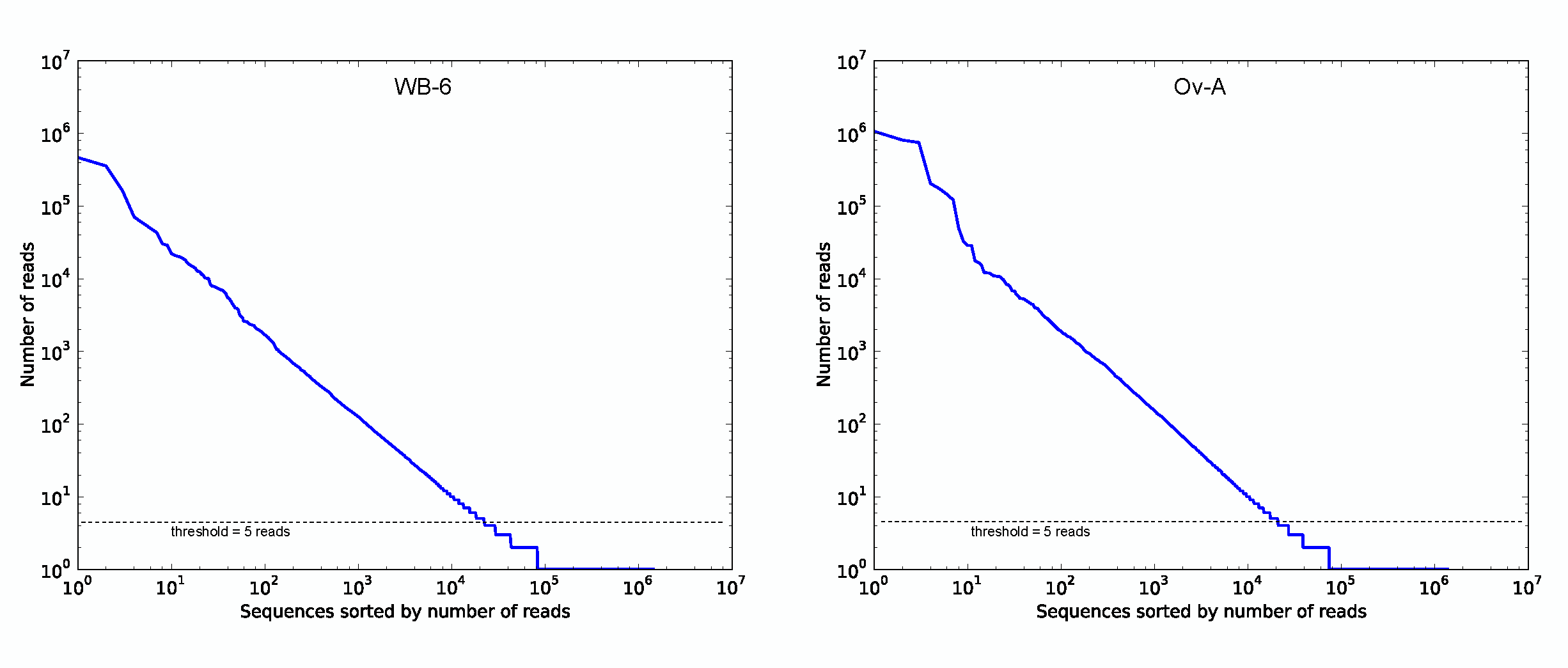

Supplement: Figure S1 — Distribution of reads per sequence for WB-6 and Ov-A libraries. (TIF) [file pone.0019350.s001.tif]

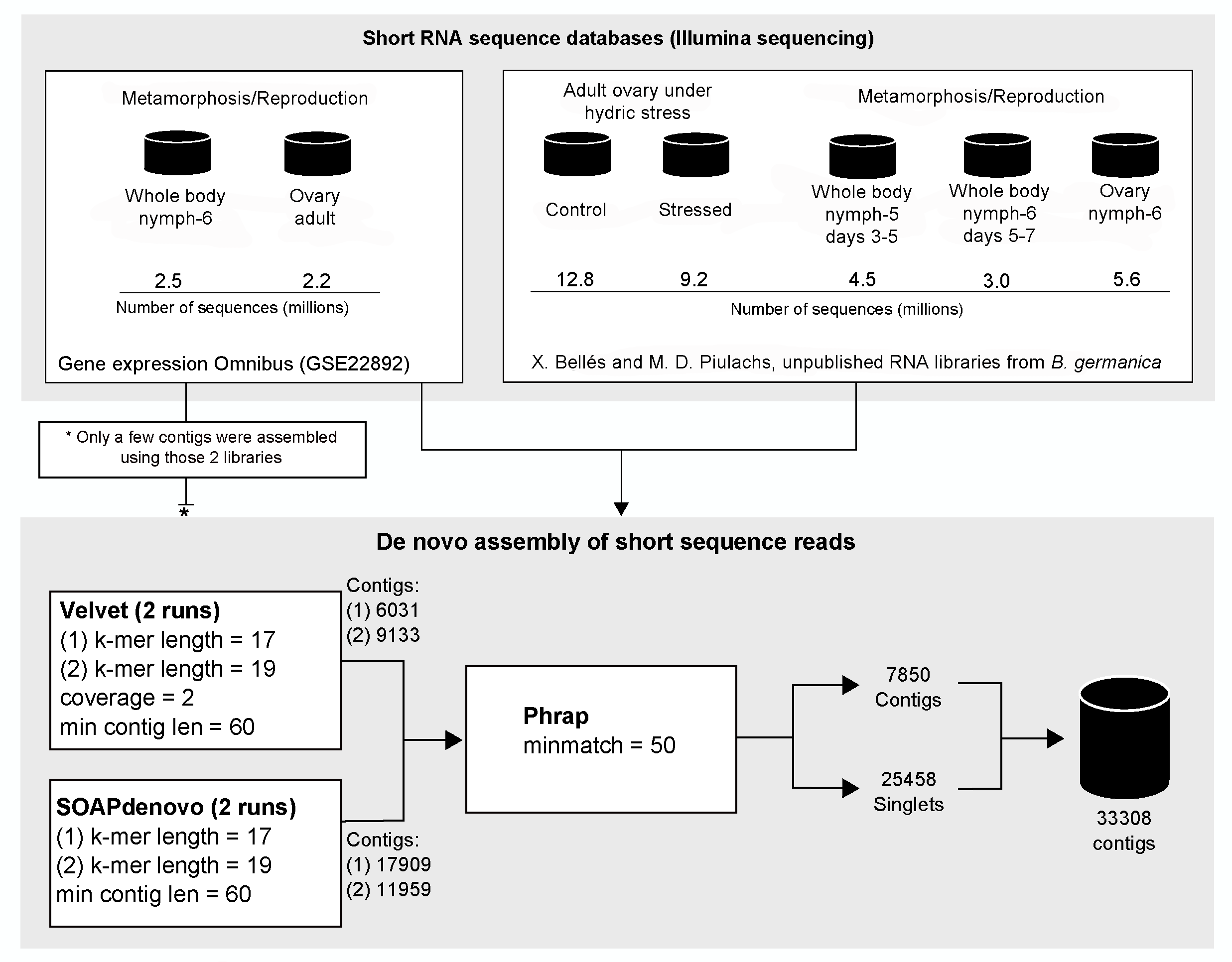

Supplement: Figure S3 — Pipeline used for assembling the sequences using Velvet, SOAPdenovo and phrap softwares. (TIF) [file pone.0019350.s003.tif]

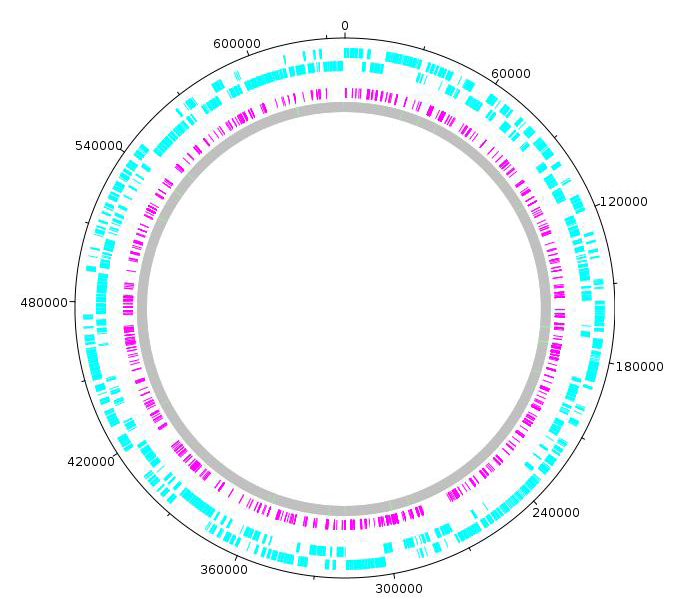

Supplement: Figure S4 — Distribution of the Blattabacterium sequences obtained in the libraries (highlighted in purple) across the Blattabacterium sp. genome (highlighted in turquoise blue). (TIF) [file pone.0019350.s004.tif]
