# Supplementary material for: Deep Sequencing of Organ- and Stage-Specific microRNAs in the Evolutionarily Basal Insect Blattella germanica (L.) (Dictyoptera, Blattellidae)
Source: PLoS One. 2011 Apr 28;6(4):e19350. doi: 10.1371/journal.pone.0019350 (PMC3084283; doi:10.1371/journal.pone.0019350)
Supplement: Text S1 — Clustering analysis of redundant miRNA candidates. (DOC) [file pone.0019350.s012.doc]

**Text S1. Clustering analysis of redundant miRNA candidates**

We used transitivity clustering method [1] to create a graph that depicts the pairwise similarity between each candidate by representing the sequences as nodes and the similarity as links. The graph is created based on alignments were perfomed by BLASTN (90% identify and 95% coverage; e-value threshold < 1e-05) and visualized using Cytoscape [2] (see Figure S2). We also used complex network methods to make a non-redundant database of novel miRNA candidates by selecting the most abundant sequences as representative of each cluster. The network analysis was performed by igraph ( http://igraph.sourceforge.net) and Python programming language (http://www.python.org). The whole similarity network is divided in 224 components being one major component composed by 1099 sequences. The major network shows virtually all possible variants for the most expressed candidate (bge_candidate1 = 4,585,181 reads), however, the modular structure of the network shows that some candidates are clustered in sub-structures (modules) which indicates they might be real sequences rather than spurious variations of bge_candidate1. We objectively measured the modularity of the large network (modularity = 0.74; [3]) and found 15 modules (see report below) which were considered as different clusters, therefore, the 1099 redundant sequences were reduced to 15 candidate sequences (Figure S2).

Only the clusters with 2 or more sequences are showed in the following report:

Cluster 14 (size: 2)

[('bge_candidate1403', 14), ('bge_candidate1340', 15)]

Cluster 39 (size: 2)

[('bge_candidate1247', 40), ('bge_candidate1179', 41)]

Cluster 44 (size: 27)

[('bge_candidate1323', 46), ('bge_candidate758', 185), ('bge_candidate593', 258), ('bge_candidate824', 261), ('bge_candidate452', 262), ('bge_candidate598', 278), ('bge_candidate920', 279), ('bge_candidate384', 446), ('bge_candidate1106', 447), ('bge_candidate1149', 448), ('bge_candidate425', 449), ('bge_candidate257', 450), ('bge_candidate1053', 503), ('bge_candidate640', 645), ('bge_candidate654', 646), ('bge_candidate704', 647), ('bge_candidate1080', 648), ('bge_candidate1299', 649), ('bge_candidate951', 650), ('bge_candidate1333', 651), ('bge_candidate153', 652), ('bge_candidate421', 666), ('bge_candidate457', 667), ('bge_candidate885', 668), ('bge_candidate1366', 669), ('bge_candidate1277', 670), ('bge_candidate146', 671)]

Cluster 51 (size: 129)

[('bge_candidate1255', 53), ('bge_candidate1368', 70), ('bge_candidate1416', 71), ('bge_candidate1079', 123), ('bge_candidate1015', 140), ('bge_candidate1084', 147), ('bge_candidate1365', 148), ('bge_candidate1103', 160), ('bge_candidate1078', 172), ('bge_candidate1232', 179), ('bge_candidate650', 181), ('bge_candidate1102', 182), ('bge_candidate1185', 205), ('bge_candidate1139', 210), ('bge_candidate583', 211), ('bge_candidate759', 218), ('bge_candidate1060', 219), ('bge_candidate547', 220), ('bge_candidate710', 221), ('bge_candidate1066', 222), ('bge_candidate546', 223), ('bge_candidate1029', 242), ('bge_candidate502', 243), ('bge_candidate952', 259), ('bge_candidate453', 260), ('bge_candidate770', 294), ('bge_candidate847', 295), ('bge_candidate566', 296), ('bge_candidate863', 297), ('bge_candidate1112', 298), ('bge_candidate1120', 299), ('bge_candidate730', 303), ('bge_candidate981', 304), ('bge_candidate391', 305), ('bge_candidate1393', 308), ('bge_candidate555', 310), ('bge_candidate445', 325), ('bge_candidate1304', 332), ('bge_candidate1346', 333), ('bge_candidate550', 334), ('bge_candidate649', 335), ('bge_candidate352', 336), ('bge_candidate429', 370), ('bge_candidate853', 371), ('bge_candidate1303', 372), ('bge_candidate1376', 373), ('bge_candidate1177', 377), ('bge_candidate621', 383), ('bge_candidate1263', 384), ('bge_candidate581', 389), ('bge_candidate300', 390), ('bge_candidate334', 403), ('bge_candidate477', 404), ('bge_candidate285', 405), ('bge_candidate1195', 426), ('bge_candidate1388', 475), ('bge_candidate470', 497), ('bge_candidate667', 522), ('bge_candidate971', 523), ('bge_candidate1141', 524), ('bge_candidate750', 525), ('bge_candidate214', 526), ('bge_candidate320', 588), ('bge_candidate788', 589), ('bge_candidate1118', 590), ('bge_candidate1035', 595), ('bge_candidate398', 596), ('bge_candidate400', 613), ('bge_candidate709', 614), ('bge_candidate957', 615), ('bge_candidate1229', 616), ('bge_candidate165', 617), ('bge_candidate350', 633), ('bge_candidate464', 634), ('bge_candidate612', 635), ('bge_candidate690', 636), ('bge_candidate720', 637), ('bge_candidate801', 638), ('bge_candidate322', 639), ('bge_candidate156', 640), ('bge_candidate251', 643), ('bge_candidate308', 695), ('bge_candidate432', 696), ('bge_candidate582', 697), ('bge_candidate1094', 698), ('bge_candidate1220', 699), ('bge_candidate357', 715), ('bge_candidate1348', 716), ('bge_candidate463', 755), ('bge_candidate1020', 756), ('bge_candidate958', 757), ('bge_candidate108', 775), ('bge_candidate505', 776), ('bge_candidate656', 777), ('bge_candidate998', 778), ('bge_candidate1428', 779), ('bge_candidate130', 798), ('bge_candidate354', 799), ('bge_candidate364', 800), ('bge_candidate409', 801), ('bge_candidate423', 802), ('bge_candidate938', 803), ('bge_candidate179', 912), ('bge_candidate317', 913), ('bge_candidate377', 914), ('bge_candidate596', 915), ('bge_candidate686', 916), ('bge_candidate833', 917), ('bge_candidate895', 918), ('bge_candidate930', 919), ('bge_candidate969', 920), ('bge_candidate1028', 921), ('bge_candidate1254', 922), ('bge_candidate58', 923), ('bge_candidate1223', 978), ('bge_candidate466', 985), ('bge_candidate1241', 987), ('bge_candidate101', 1036), ('bge_candidate123', 1037), ('bge_candidate271', 1038), ('bge_candidate305', 1039), ('bge_candidate549', 1040), ('bge_candidate577', 1041), ('bge_candidate594', 1042), ('bge_candidate653', 1043), ('bge_candidate745', 1044), ('bge_candidate1375', 1045), ('bge_candidate1392', 1046), ('bge_candidate28', 1047)]

Cluster 57 (size: 4)

[('bge_candidate1383', 59), ('bge_candidate877', 142), ('bge_candidate1096', 143), ('bge_candidate752', 144)]

Cluster 59 (size: 2)

[('bge_candidate1414', 61), ('bge_candidate1087', 62)]

Cluster 65 (size: 213)

[('bge_candidate1361', 68), ('bge_candidate1272', 97), ('bge_candidate1069', 122), ('bge_candidate1100', 158), ('bge_candidate1233', 187), ('bge_candidate1242', 188), ('bge_candidate1362', 189), ('bge_candidate992', 199), ('bge_candidate965', 208), ('bge_candidate584', 209), ('bge_candidate571', 214), ('bge_candidate1180', 241), ('bge_candidate1296', 267), ('bge_candidate1150', 270), ('bge_candidate433', 271), ('bge_candidate889', 273), ('bge_candidate716', 280), ('bge_candidate387', 306), ('bge_candidate1235', 315), ('bge_candidate374', 316), ('bge_candidate903', 322), ('bge_candidate1307', 323), ('bge_candidate537', 324), ('bge_candidate1041', 326), ('bge_candidate401', 327), ('bge_candidate961', 330), ('bge_candidate1426', 348), ('bge_candidate337', 351), ('bge_candidate506', 360), ('bge_candidate600', 361), ('bge_candidate1381', 362), ('bge_candidate892', 363), ('bge_candidate849', 364), ('bge_candidate1399', 369), ('bge_candidate575', 374), ('bge_candidate367', 375), ('bge_candidate318', 376), ('bge_candidate699', 391), ('bge_candidate1269', 400), ('bge_candidate636', 406), ('bge_candidate580', 418), ('bge_candidate827', 419), ('bge_candidate767', 420), ('bge_candidate275', 421), ('bge_candidate1377', 434), ('bge_candidate634', 444), ('bge_candidate820', 445), ('bge_candidate1374', 451), ('bge_candidate1067', 458), ('bge_candidate1292', 459), ('bge_candidate245', 460), ('bge_candidate459', 471), ('bge_candidate1054', 472), ('bge_candidate1062', 473), ('bge_candidate1050', 478), ('bge_candidate365', 479), ('bge_candidate1321', 480), ('bge_candidate1032', 481), ('bge_candidate430', 489), ('bge_candidate1031', 490), ('bge_candidate708', 499), ('bge_candidate1183', 500), ('bge_candidate309', 501), ('bge_candidate818', 502), ('bge_candidate1200', 504), ('bge_candidate222', 505), ('bge_candidate417', 508), ('bge_candidate635', 509), ('bge_candidate1423', 510), ('bge_candidate250', 512), ('bge_candidate481', 513), ('bge_candidate717', 515), ('bge_candidate1270', 517), ('bge_candidate1317', 518), ('bge_candidate319', 520), ('bge_candidate216', 521), ('bge_candidate996', 534), ('bge_candidate1331', 550), ('bge_candidate942', 559), ('bge_candidate442', 585), ('bge_candidate1004', 594), ('bge_candidate441', 597), ('bge_candidate713', 598), ('bge_candidate867', 599), ('bge_candidate366', 604), ('bge_candidate631', 607), ('bge_candidate714', 608), ('bge_candidate982', 609), ('bge_candidate568', 627), ('bge_candidate808', 675), ('bge_candidate946', 676), ('bge_candidate1251', 677), ('bge_candidate299', 679), ('bge_candidate369', 680), ('bge_candidate779', 681), ('bge_candidate813', 682), ('bge_candidate825', 683), ('bge_candidate1047', 688), ('bge_candidate834', 705), ('bge_candidate1353', 706), ('bge_candidate1142', 707), ('bge_candidate388', 708), ('bge_candidate910', 709), ('bge_candidate219', 733), ('bge_candidate225', 734), ('bge_candidate330', 735), ('bge_candidate455', 736), ('bge_candidate539', 737), ('bge_candidate1012', 738), ('bge_candidate1057', 739), ('bge_candidate172', 740), ('bge_candidate335', 741), ('bge_candidate482', 742), ('bge_candidate948', 744), ('bge_candidate113', 745), ('bge_candidate126', 750), ('bge_candidate643', 751), ('bge_candidate881', 752), ('bge_candidate545', 754), ('bge_candidate670', 758), ('bge_candidate780', 759), ('bge_candidate904', 760), ('bge_candidate994', 761), ('bge_candidate1147', 762), ('bge_candidate972', 763), ('bge_candidate669', 764), ('bge_candidate174', 792), ('bge_candidate181', 793), ('bge_candidate422', 795), ('bge_candidate510', 796), ('bge_candidate799', 797), ('bge_candidate323', 804), ('bge_candidate689', 805), ('bge_candidate1206', 806), ('bge_candidate614', 807), ('bge_candidate544', 808), ('bge_candidate93', 809), ('bge_candidate107', 868), ('bge_candidate208', 869), ('bge_candidate638', 870), ('bge_candidate835', 871), ('bge_candidate1030', 872), ('bge_candidate1130', 873), ('bge_candidate1396', 874), ('bge_candidate540', 875), ('bge_candidate622', 876), ('bge_candidate1395', 877), ('bge_candidate68', 878), ('bge_candidate168', 893), ('bge_candidate485', 894), ('bge_candidate648', 895), ('bge_candidate434', 897), ('bge_candidate790', 898), ('bge_candidate1228', 899), ('bge_candidate115', 905), ('bge_candidate142', 906), ('bge_candidate259', 907), ('bge_candidate290', 908), ('bge_candidate358', 909), ('bge_candidate462', 910), ('bge_candidate671', 911), ('bge_candidate236', 933), ('bge_candidate503', 934), ('bge_candidate1168', 935), ('bge_candidate900', 936), ('bge_candidate55', 937), ('bge_candidate109', 952), ('bge_candidate173', 953), ('bge_candidate231', 954), ('bge_candidate518', 955), ('bge_candidate595', 956), ('bge_candidate888', 957), ('bge_candidate1048', 958), ('bge_candidate141', 974), ('bge_candidate363', 976), ('bge_candidate760', 977), ('bge_candidate1370', 979), ('bge_candidate511', 986), ('bge_candidate47', 1014), ('bge_candidate59', 1015), ('bge_candidate198', 1016), ('bge_candidate218', 1017), ('bge_candidate235', 1018), ('bge_candidate553', 1019), ('bge_candidate763', 1020), ('bge_candidate862', 1021), ('bge_candidate1406', 1022), ('bge_candidate32', 1025), ('bge_candidate132', 1026), ('bge_candidate256', 1027), ('bge_candidate289', 1028), ('bge_candidate324', 1029), ('bge_candidate851', 1030), ('bge_candidate926', 1031), ('bge_candidate806', 1032), ('bge_candidate30', 1033), ('bge_candidate61', 1105), ('bge_candidate96', 1106), ('bge_candidate241', 1108), ('bge_candidate315', 1109), ('bge_candidate338', 1110), ('bge_candidate637', 1111), ('bge_candidate956', 1112), ('bge_candidate42', 1113), ('bge_candidate95', 1114), ('bge_candidate18', 1115), ('bge_candidate62', 1152), ('bge_candidate331', 1153), ('bge_candidate602', 1154), ('bge_candidate1131', 1155), ('bge_candidate127', 1156), ('bge_candidate1294', 1157), ('bge_candidate13', 1158)]

Cluster 67 (size: 7)

[('bge_candidate1088', 72), ('bge_candidate1025', 118), ('bge_candidate685', 244), ('bge_candidate705', 245), ('bge_candidate830', 246), ('bge_candidate854', 247), ('bge_candidate498', 248)]

Cluster 72 (size: 5)

[('bge_candidate1385', 77), ('bge_candidate1002', 78), ('bge_candidate1410', 167), ('bge_candidate688', 392), ('bge_candidate295', 393)]

Cluster 76 (size: 3)

[('bge_candidate980', 82), ('bge_candidate1038', 276), ('bge_candidate424', 277)]

Cluster 87 (size: 2)

[('bge_candidate1266', 93), ('bge_candidate937', 94)]

Cluster 89 (size: 87)

[('bge_candidate928', 96), ('bge_candidate915', 216), ('bge_candidate1037', 331), ('bge_candidate1357', 401), ('bge_candidate1315', 498), ('bge_candidate897', 506), ('bge_candidate934', 507), ('bge_candidate557', 528), ('bge_candidate724', 529), ('bge_candidate1042', 530), ('bge_candidate526', 553), ('bge_candidate646', 554), ('bge_candidate531', 574), ('bge_candidate536', 575), ('bge_candidate1314', 576), ('bge_candidate1287', 601), ('bge_candidate221', 603), ('bge_candidate385', 605), ('bge_candidate587', 606), ('bge_candidate195', 625), ('bge_candidate283', 626), ('bge_candidate664', 628), ('bge_candidate419', 644), ('bge_candidate169', 672), ('bge_candidate1191', 673), ('bge_candidate238', 678), ('bge_candidate160', 686), ('bge_candidate507', 687), ('bge_candidate1128', 731), ('bge_candidate1013', 753), ('bge_candidate212', 770), ('bge_candidate909', 773), ('bge_candidate1421', 774), ('bge_candidate223', 794), ('bge_candidate188', 838), ('bge_candidate707', 840), ('bge_candidate80', 841), ('bge_candidate137', 861), ('bge_candidate325', 862), ('bge_candidate1166', 863), ('bge_candidate652', 867), ('bge_candidate224', 896), ('bge_candidate229', 900), ('bge_candidate288', 901), ('bge_candidate1249', 903), ('bge_candidate105', 904), ('bge_candidate145', 948), ('bge_candidate504', 950), ('bge_candidate1328', 969), ('bge_candidate226', 975), ('bge_candidate139', 980), ('bge_candidate171', 981), ('bge_candidate418', 982), ('bge_candidate659', 983), ('bge_candidate1083', 984), ('bge_candidate280', 1088), ('bge_candidate154', 1107), ('bge_candidate804', 1161), ('bge_candidate1190', 1162), ('bge_candidate60', 1164), ('bge_candidate71', 1165), ('bge_candidate248', 1166), ('bge_candidate314', 1167), ('bge_candidate1306', 1172), ('bge_candidate194', 1246), ('bge_candidate389', 1253), ('bge_candidate668', 1258), ('bge_candidate967', 1261), ('bge_candidate1114', 1263), ('bge_candidate1373', 1266), ('bge_candidate450', 1267), ('bge_candidate792', 1268), ('bge_candidate1298', 1269), ('bge_candidate684', 1270), ('bge_candidate11', 1296), ('bge_candidate40', 1297), ('bge_candidate252', 1302), ('bge_candidate839', 1317), ('bge_candidate1210', 1323), ('bge_candidate104', 1364), ('bge_candidate307', 1368), ('bge_candidate76', 1402), ('bge_candidate87', 1403), ('bge_candidate478', 1413), ('bge_candidate675', 1414), ('bge_candidate1056', 1416), ('bge_candidate1061', 1417)]

Cluster 93 (size: 4)

[('bge_candidate1425', 101), ('bge_candidate856', 130), ('bge_candidate918', 131), ('bge_candidate782', 132)]

Cluster 103 (size: 2)

[('bge_candidate1283', 111), ('bge_candidate868', 112)]

Cluster 104 (size: 2)

[('bge_candidate1291', 113), ('bge_candidate865', 114)]

Cluster 105 (size: 2)

[('bge_candidate1245', 115), ('bge_candidate858', 116)]

Cluster 109 (size: 5)

[('bge_candidate1281', 121), ('bge_candidate712', 200), ('bge_candidate836', 201), ('bge_candidate1113', 202), ('bge_candidate599', 203)]

Cluster 120 (size: 6)

[('bge_candidate1043', 137), ('bge_candidate766', 145), ('bge_candidate1363', 146), ('bge_candidate751', 155), ('bge_candidate932', 156), ('bge_candidate718', 157)]

Cluster 121 (size: 2)

[('bge_candidate811', 138), ('bge_candidate764', 139)]

Cluster 130 (size: 4)

[('bge_candidate968', 161), ('bge_candidate679', 551), ('bge_candidate196', 552), ('bge_candidate706', 743)]

Cluster 133 (size: 4)

[('bge_candidate698', 164), ('bge_candidate794', 264), ('bge_candidate887', 265), ('bge_candidate443', 266)]

Cluster 136 (size: 134)

[('bge_candidate1367', 168), ('bge_candidate1318', 232), ('bge_candidate454', 274), ('bge_candidate426', 275), ('bge_candidate381', 309), ('bge_candidate376', 311), ('bge_candidate480', 312), ('bge_candidate735', 313), ('bge_candidate375', 314), ('bge_candidate359', 328), ('bge_candidate348', 338), ('bge_candidate1162', 366), ('bge_candidate328', 367), ('bge_candidate311', 382), ('bge_candidate1354', 385), ('bge_candidate304', 386), ('bge_candidate1221', 395), ('bge_candidate1324', 396), ('bge_candidate293', 397), ('bge_candidate1246', 411), ('bge_candidate1335', 412), ('bge_candidate1199', 413), ('bge_candidate277', 414), ('bge_candidate802', 423), ('bge_candidate1055', 424), ('bge_candidate272', 425), ('bge_candidate1216', 430), ('bge_candidate523', 431), ('bge_candidate267', 432), ('bge_candidate1276', 433), ('bge_candidate1271', 435), ('bge_candidate266', 436), ('bge_candidate1115', 440), ('bge_candidate261', 441), ('bge_candidate1186', 456), ('bge_candidate246', 457), ('bge_candidate520', 462), ('bge_candidate999', 463), ('bge_candidate1316', 464), ('bge_candidate469', 465), ('bge_candidate313', 466), ('bge_candidate645', 467), ('bge_candidate1301', 468), ('bge_candidate1044', 469), ('bge_candidate242', 470), ('bge_candidate913', 484), ('bge_candidate1350', 485), ('bge_candidate1394', 486), ('bge_candidate1204', 487), ('bge_candidate232', 488), ('bge_candidate1302', 492), ('bge_candidate736', 493), ('bge_candidate228', 494), ('bge_candidate610', 514), ('bge_candidate1157', 516), ('bge_candidate1322', 519), ('bge_candidate1264', 535), ('bge_candidate1391', 536), ('bge_candidate207', 537), ('bge_candidate1005', 538), ('bge_candidate1007', 539), ('bge_candidate1017', 540), ('bge_candidate990', 541), ('bge_candidate206', 542), ('bge_candidate723', 543), ('bge_candidate726', 544), ('bge_candidate1389', 545), ('bge_candidate1098', 547), ('bge_candidate719', 548), ('bge_candidate199', 549), ('bge_candidate576', 555), ('bge_candidate687', 556), ('bge_candidate776', 557), ('bge_candidate902', 558), ('bge_candidate983', 560), ('bge_candidate1143', 561), ('bge_candidate1198', 562), ('bge_candidate1253', 563), ('bge_candidate732', 564), ('bge_candidate193', 565), ('bge_candidate1008', 567), ('bge_candidate1151', 568), ('bge_candidate1224', 569), ('bge_candidate1108', 570), ('bge_candidate191', 571), ('bge_candidate816', 577), ('bge_candidate1068', 578), ('bge_candidate1121', 579), ('bge_candidate186', 580), ('bge_candidate243', 583), ('bge_candidate1018', 602), ('bge_candidate677', 618), ('bge_candidate875', 619), ('bge_candidate1105', 620), ('bge_candidate1230', 621), ('bge_candidate1237', 622), ('bge_candidate1016', 623), ('bge_candidate163', 624), ('bge_candidate530', 811), ('bge_candidate700', 812), ('bge_candidate817', 813), ('bge_candidate1023', 814), ('bge_candidate1236', 815), ('bge_candidate170', 816), ('bge_candidate91', 817), ('bge_candidate203', 820), ('bge_candidate353', 824), ('bge_candidate783', 825), ('bge_candidate905', 826), ('bge_candidate1040', 827), ('bge_candidate1178', 828), ('bge_candidate83', 829), ('bge_candidate516', 847), ('bge_candidate796', 848), ('bge_candidate943', 849), ('bge_candidate991', 850), ('bge_candidate1034', 851), ('bge_candidate75', 852), ('bge_candidate183', 1001), ('bge_candidate184', 1002), ('bge_candidate461', 1003), ('bge_candidate86', 1004), ('bge_candidate565', 1049), ('bge_candidate569', 1050), ('bge_candidate746', 1051), ('bge_candidate1000', 1052), ('bge_candidate509', 1053), ('bge_candidate26', 1064), ('bge_candidate35', 1065), ('bge_candidate512', 1066), ('bge_candidate786', 1067), ('bge_candidate795', 1068), ('bge_candidate201', 1069), ('bge_candidate24', 1070)]

Cluster 138 (size: 26)

[('bge_candidate1422', 170), ('bge_candidate1135', 272), ('bge_candidate1218', 288), ('bge_candidate1344', 378), ('bge_candidate676', 407), ('bge_candidate471', 408), ('bge_candidate1387', 409), ('bge_candidate844', 410), ('bge_candidate282', 422), ('bge_candidate1027', 496), ('bge_candidate273', 720), ('bge_candidate278', 721), ('bge_candidate408', 722), ('bge_candidate563', 723), ('bge_candidate742', 724), ('bge_candidate793', 725), ('bge_candidate1014', 726), ('bge_candidate1036', 727), ('bge_candidate431', 728), ('bge_candidate119', 729), ('bge_candidate696', 786), ('bge_candidate316', 839), ('bge_candidate449', 902), ('bge_candidate674', 951), ('bge_candidate606', 1168), ('bge_candidate499', 1254)]

Cluster 141 (size: 144)

[('bge_candidate1280', 174), ('bge_candidate1203', 213), ('bge_candidate1412', 217), ('bge_candidate1258', 230), ('bge_candidate1372', 257), ('bge_candidate1358', 268), ('bge_candidate873', 289), ('bge_candidate1248', 292), ('bge_candidate1250', 293), ('bge_candidate749', 321), ('bge_candidate785', 339), ('bge_candidate1110', 340), ('bge_candidate1284', 343), ('bge_candidate475', 353), ('bge_candidate880', 354), ('bge_candidate777', 398), ('bge_candidate1278', 399), ('bge_candidate791', 427), ('bge_candidate572', 437), ('bge_candidate1144', 438), ('bge_candidate552', 439), ('bge_candidate347', 452), ('bge_candidate633', 453), ('bge_candidate843', 477), ('bge_candidate487', 482), ('bge_candidate740', 491), ('bge_candidate489', 495), ('bge_candidate662', 531), ('bge_candidate861', 532), ('bge_candidate765', 546), ('bge_candidate521', 581), ('bge_candidate1188', 582), ('bge_candidate772', 587), ('bge_candidate682', 592), ('bge_candidate893', 593), ('bge_candidate1176', 600), ('bge_candidate396', 653), ('bge_candidate702', 654), ('bge_candidate573', 655), ('bge_candidate733', 656), ('bge_candidate151', 657), ('bge_candidate927', 658), ('bge_candidate368', 663), ('bge_candidate451', 664), ('bge_candidate1159', 674), ('bge_candidate695', 684), ('bge_candidate333', 685), ('bge_candidate542', 689), ('bge_candidate579', 691), ('bge_candidate611', 692), ('bge_candidate683', 700), ('bge_candidate1405', 701), ('bge_candidate152', 710), ('bge_candidate407', 711), ('bge_candidate1310', 765), ('bge_candidate210', 766), ('bge_candidate270', 767), ('bge_candidate743', 768), ('bge_candidate402', 771), ('bge_candidate891', 772), ('bge_candidate437', 785), ('bge_candidate954', 787), ('bge_candidate625', 810), ('bge_candidate950', 821), ('bge_candidate936', 823), ('bge_candidate1274', 830), ('bge_candidate440', 831), ('bge_candidate513', 845), ('bge_candidate678', 856), ('bge_candidate907', 860), ('bge_candidate497', 866), ('bge_candidate342', 880), ('bge_candidate642', 881), ('bge_candidate1420', 882), ('bge_candidate882', 883), ('bge_candidate428', 885), ('bge_candidate738', 886), ('bge_candidate1360', 887), ('bge_candidate106', 888), ('bge_candidate264', 889), ('bge_candidate1174', 890), ('bge_candidate382', 927), ('bge_candidate416', 928), ('bge_candidate175', 930), ('bge_candidate474', 931), ('bge_candidate1268', 932), ('bge_candidate215', 949), ('bge_candidate185', 960), ('bge_candidate360', 962), ('bge_candidate500', 988), ('bge_candidate230', 989), ('bge_candidate133', 990), ('bge_candidate345', 996), ('bge_candidate346', 997), ('bge_candidate262', 1009), ('bge_candidate438', 1010), ('bge_candidate140', 1080), ('bge_candidate202', 1081), ('bge_candidate1089', 1117), ('bge_candidate1418', 1118), ('bge_candidate784', 1137), ('bge_candidate963', 1138), ('bge_candidate1173', 1139), ('bge_candidate1212', 1140), ('bge_candidate448', 1141), ('bge_candidate1325', 1142), ('bge_candidate490', 1143), ('bge_candidate1049', 1144), ('bge_candidate1257', 1145), ('bge_candidate1339', 1146), ('bge_candidate64', 1147), ('bge_candidate484', 1148), ('bge_candidate789', 1149), ('bge_candidate1192', 1150), ('bge_candidate468', 1151), ('bge_candidate303', 1159), ('bge_candidate298', 1160), ('bge_candidate1184', 1171), ('bge_candidate379', 1193), ('bge_candidate439', 1195), ('bge_candidate129', 1208), ('bge_candidate197', 1211), ('bge_candidate560', 1217), ('bge_candidate1267', 1224), ('bge_candidate85', 1227), ('bge_candidate92', 1228), ('bge_candidate269', 1249), ('bge_candidate355', 1250), ('bge_candidate554', 1256), ('bge_candidate1154', 1264), ('bge_candidate67', 1278), ('bge_candidate78', 1280), ('bge_candidate1125', 1290), ('bge_candidate467', 1307), ('bge_candidate479', 1308), ('bge_candidate693', 1315), ('bge_candidate56', 1339), ('bge_candidate82', 1340), ('bge_candidate205', 1366), ('bge_candidate274', 1367), ('bge_candidate14', 1386), ('bge_candidate15', 1387), ('bge_candidate54', 1398), ('bge_candidate66', 1400)]

Cluster 142 (size: 2)

[('bge_candidate1259', 175), ('bge_candidate661', 176)]

Cluster 143 (size: 14)

[('bge_candidate995', 177), ('bge_candidate841', 178), ('bge_candidate872', 212), ('bge_candidate1095', 379), ('bge_candidate578', 380), ('bge_candidate721', 381), ('bge_candidate415', 611), ('bge_candidate691', 612), ('bge_candidate39', 1071), ('bge_candidate166', 1072), ('bge_candidate312', 1073), ('bge_candidate655', 1074), ('bge_candidate1215', 1075), ('bge_candidate23', 1076)]

Cluster 154 (size: 2)

[('bge_candidate1219', 196), ('bge_candidate607', 197)]

Cluster 169 (size: 7)

[('bge_candidate1155', 235), ('bge_candidate1408', 236), ('bge_candidate1202', 237), ('bge_candidate515', 238), ('bge_candidate797', 250), ('bge_candidate823', 251), ('bge_candidate495', 252)]

Cluster 170 (size: 2)

[('bge_candidate1076', 239), ('bge_candidate514', 240)]

Cluster 175 (size: 14)

[('bge_candidate605', 256), ('bge_candidate1070', 337), ('bge_candidate240', 1125), ('bge_candidate349', 1126), ('bge_candidate472', 1127), ('bge_candidate473', 1128), ('bge_candidate543', 1129), ('bge_candidate800', 1130), ('bge_candidate874', 1131), ('bge_candidate1137', 1132), ('bge_candidate1342', 1133), ('bge_candidate1386', 1134), ('bge_candidate1074', 1135), ('bge_candidate16', 1136)]

Cluster 178 (size: 8)

[('bge_candidate931', 281), ('bge_candidate1351', 282), ('bge_candidate414', 283), ('bge_candidate405', 355), ('bge_candidate739', 356), ('bge_candidate1413', 357), ('bge_candidate744', 358), ('bge_candidate332', 359)]

Cluster 179 (size: 4)

[('bge_candidate427', 284), ('bge_candidate483', 285), ('bge_candidate1059', 286), ('bge_candidate413', 287)]

Cluster 181 (size: 180)

[('bge_candidate1411', 291), ('bge_candidate1327', 394), ('bge_candidate624', 476), ('bge_candidate1163', 483), ('bge_candidate819', 533), ('bge_candidate1326', 572), ('bge_candidate528', 586), ('bge_candidate878', 631), ('bge_candidate255', 662), ('bge_candidate1309', 702), ('bge_candidate986', 703), ('bge_candidate1239', 704), ('bge_candidate494', 712), ('bge_candidate826', 769), ('bge_candidate1275', 788), ('bge_candidate945', 819), ('bge_candidate925', 822), ('bge_candidate524', 842), ('bge_candidate1222', 843), ('bge_candidate898', 844), ('bge_candidate613', 846), ('bge_candidate964', 853), ('bge_candidate1126', 854), ('bge_candidate458', 855), ('bge_candidate890', 857), ('bge_candidate1400', 879), ('bge_candidate1402', 884), ('bge_candidate148', 924), ('bge_candidate1288', 925), ('bge_candidate234', 926), ('bge_candidate1136', 929), ('bge_candidate286', 961), ('bge_candidate1082', 963), ('bge_candidate1201', 964), ('bge_candidate1127', 965), ('bge_candidate641', 971), ('bge_candidate1336', 972), ('bge_candidate301', 991), ('bge_candidate1227', 992), ('bge_candidate773', 993), ('bge_candidate291', 994), ('bge_candidate237', 995), ('bge_candidate564', 998), ('bge_candidate953', 999), ('bge_candidate1122', 1000), ('bge_candidate263', 1005), ('bge_candidate741', 1006), ('bge_candidate805', 1007), ('bge_candidate180', 1008), ('bge_candidate672', 1011), ('bge_candidate734', 1012), ('bge_candidate1305', 1013), ('bge_candidate866', 1023), ('bge_candidate876', 1024), ('bge_candidate810', 1057), ('bge_candidate125', 1077), ('bge_candidate551', 1078), ('bge_candidate57', 1079), ('bge_candidate254', 1082), ('bge_candidate812', 1084), ('bge_candidate559', 1120), ('bge_candidate822', 1121), ('bge_candidate1046', 1122), ('bge_candidate1145', 1123), ('bge_candidate1207', 1124), ('bge_candidate1065', 1169), ('bge_candidate1182', 1173), ('bge_candidate620', 1174), ('bge_candidate632', 1175), ('bge_candidate657', 1176), ('bge_candidate761', 1177), ('bge_candidate860', 1178), ('bge_candidate619', 1180), ('bge_candidate940', 1181), ('bge_candidate1398', 1182), ('bge_candidate728', 1184), ('bge_candidate831', 1185), ('bge_candidate838', 1186), ('bge_candidate17', 1192), ('bge_candidate465', 1196), ('bge_candidate798', 1203), ('bge_candidate857', 1204), ('bge_candidate922', 1205), ('bge_candidate1343', 1206), ('bge_candidate128', 1207), ('bge_candidate138', 1209), ('bge_candidate144', 1210), ('bge_candidate541', 1212), ('bge_candidate1001', 1213), ('bge_candidate1099', 1214), ('bge_candidate1132', 1215), ('bge_candidate296', 1216), ('bge_candidate870', 1219), ('bge_candidate36', 1226), ('bge_candidate729', 1232), ('bge_candidate975', 1234), ('bge_candidate1153', 1235), ('bge_candidate1226', 1236), ('bge_candidate1243', 1237), ('bge_candidate99', 1243), ('bge_candidate176', 1245), ('bge_candidate211', 1247), ('bge_candidate233', 1248), ('bge_candidate370', 1252), ('bge_candidate846', 1259), ('bge_candidate914', 1260), ('bge_candidate974', 1262), ('bge_candidate1158', 1265), ('bge_candidate393', 1273), ('bge_candidate807', 1274), ('bge_candidate1211', 1275), ('bge_candidate1384', 1276), ('bge_candidate45', 1277), ('bge_candidate73', 1279), ('bge_candidate79', 1281), ('bge_candidate204', 1282), ('bge_candidate220', 1283), ('bge_candidate292', 1284), ('bge_candidate294', 1285), ('bge_candidate864', 1286), ('bge_candidate984', 1287), ('bge_candidate1019', 1288), ('bge_candidate1045', 1289), ('bge_candidate1133', 1291), ('bge_candidate837', 1293), ('bge_candidate1081', 1294), ('bge_candidate265', 1295), ('bge_candidate561', 1311), ('bge_candidate574', 1312), ('bge_candidate590', 1313), ('bge_candidate756', 1316), ('bge_candidate845', 1318), ('bge_candidate966', 1319), ('bge_candidate1279', 1322), ('bge_candidate143', 1324), ('bge_candidate748', 1328), ('bge_candidate814', 1329), ('bge_candidate894', 1330), ('bge_candidate935', 1331), ('bge_candidate1256', 1332), ('bge_candidate1341', 1333), ('bge_candidate1359', 1334), ('bge_candidate1419', 1335), ('bge_candidate22', 1336), ('bge_candidate34', 1337), ('bge_candidate37', 1338), ('bge_candidate89', 1341), ('bge_candidate258', 1342), ('bge_candidate279', 1343), ('bge_candidate281', 1344), ('bge_candidate362', 1345), ('bge_candidate525', 1346), ('bge_candidate623', 1347), ('bge_candidate658', 1348), ('bge_candidate681', 1349), ('bge_candidate753', 1350), ('bge_candidate769', 1351), ('bge_candidate923', 1352), ('bge_candidate1313', 1353), ('bge_candidate501', 1354), ('bge_candidate879', 1355), ('bge_candidate916', 1356), ('bge_candidate190', 1357), ('bge_candidate6', 1358), ('bge_candidate114', 1365), ('bge_candidate1338', 1377), ('bge_candidate2', 1378), ('bge_candidate4', 1380), ('bge_candidate8', 1382), ('bge_candidate10', 1384), ('bge_candidate31', 1391), ('bge_candidate33', 1392), ('bge_candidate38', 1393), ('bge_candidate50', 1396), ('bge_candidate53', 1397), ('bge_candidate65', 1399), ('bge_candidate74', 1401), ('bge_candidate88', 1404), ('bge_candidate120', 1407), ('bge_candidate162', 1408)]

Cluster 182 (size: 5)

[('bge_candidate604', 300), ('bge_candidate340', 346), ('bge_candidate397', 347), ('bge_candidate768', 349), ('bge_candidate339', 350)]

Cluster 187 (size: 3)

[('bge_candidate1024', 318), ('bge_candidate1320', 319), ('bge_candidate372', 320)]

Cluster 191 (size: 2)

[('bge_candidate447', 344), ('bge_candidate341', 345)]

Cluster 195 (size: 2)

[('bge_candidate1160', 387), ('bge_candidate302', 388)]

Cluster 197 (size: 3)

[('bge_candidate326', 415), ('bge_candidate591', 416), ('bge_candidate276', 417)]

Cluster 198 (size: 2)

[('bge_candidate1092', 428), ('bge_candidate268', 429)]

Cluster 199 (size: 2)

[('bge_candidate411', 442), ('bge_candidate260', 443)]

Cluster 201 (size: 130)

[('bge_candidate588', 455), ('bge_candidate1273', 630), ('bge_candidate1073', 632), ('bge_candidate955', 659), ('bge_candidate209', 858), ('bge_candidate562', 859), ('bge_candidate227', 865), ('bge_candidate1117', 891), ('bge_candidate1409', 892), ('bge_candidate1209', 959), ('bge_candidate1297', 968), ('bge_candidate601', 970), ('bge_candidate1355', 973), ('bge_candidate755', 1034), ('bge_candidate1170', 1035), ('bge_candidate158', 1048), ('bge_candidate762', 1054), ('bge_candidate351', 1055), ('bge_candidate589', 1056), ('bge_candidate1011', 1058), ('bge_candidate1134', 1059), ('bge_candidate1217', 1060), ('bge_candidate1371', 1061), ('bge_candidate1382', 1062), ('bge_candidate404', 1063), ('bge_candidate1285', 1083), ('bge_candidate629', 1085), ('bge_candidate908', 1086), ('bge_candidate933', 1087), ('bge_candidate321', 1089), ('bge_candidate821', 1090), ('bge_candidate850', 1091), ('bge_candidate960', 1092), ('bge_candidate987', 1093), ('bge_candidate1172', 1094), ('bge_candidate249', 1095), ('bge_candidate403', 1096), ('bge_candidate460', 1116), ('bge_candidate558', 1119), ('bge_candidate48', 1163), ('bge_candidate1164', 1170), ('bge_candidate1337', 1179), ('bge_candidate476', 1183), ('bge_candidate976', 1187), ('bge_candidate1010', 1188), ('bge_candidate1169', 1189), ('bge_candidate1181', 1190), ('bge_candidate1289', 1191), ('bge_candidate420', 1194), ('bge_candidate754', 1197), ('bge_candidate778', 1198), ('bge_candidate627', 1199), ('bge_candidate852', 1200), ('bge_candidate1208', 1201), ('bge_candidate803', 1202), ('bge_candidate665', 1218), ('bge_candidate884', 1220), ('bge_candidate997', 1221), ('bge_candidate1064', 1222), ('bge_candidate1090', 1223), ('bge_candidate1308', 1225), ('bge_candidate117', 1229), ('bge_candidate178', 1230), ('bge_candidate392', 1231), ('bge_candidate848', 1233), ('bge_candidate1417', 1238), ('bge_candidate380', 1239), ('bge_candidate977', 1240), ('bge_candidate1345', 1241), ('bge_candidate1026', 1242), ('bge_candidate135', 1244), ('bge_candidate361', 1251), ('bge_candidate517', 1255), ('bge_candidate663', 1257), ('bge_candidate1252', 1271), ('bge_candidate5', 1272), ('bge_candidate1356', 1292), ('bge_candidate69', 1298), ('bge_candidate150', 1299), ('bge_candidate157', 1300), ('bge_candidate247', 1301), ('bge_candidate297', 1303), ('bge_candidate306', 1304), ('bge_candidate371', 1305), ('bge_candidate386', 1306), ('bge_candidate488', 1309), ('bge_candidate493', 1310), ('bge_candidate615', 1314), ('bge_candidate988', 1320), ('bge_candidate1189', 1321), ('bge_candidate310', 1325), ('bge_candidate456', 1326), ('bge_candidate567', 1327), ('bge_candidate20', 1359), ('bge_candidate63', 1360), ('bge_candidate72', 1361), ('bge_candidate77', 1362), ('bge_candidate84', 1363), ('bge_candidate399', 1369), ('bge_candidate412', 1370), ('bge_candidate444', 1371), ('bge_candidate556', 1372), ('bge_candidate715', 1373), ('bge_candidate1063', 1374), ('bge_candidate1175', 1375), ('bge_candidate1238', 1376), ('bge_candidate3', 1379), ('bge_candidate7', 1381), ('bge_candidate9', 1383), ('bge_candidate12', 1385), ('bge_candidate25', 1388), ('bge_candidate27', 1389), ('bge_candidate29', 1390), ('bge_candidate41', 1394), ('bge_candidate46', 1395), ('bge_candidate94', 1405), ('bge_candidate103', 1406), ('bge_candidate164', 1409), ('bge_candidate187', 1410), ('bge_candidate378', 1411), ('bge_candidate436', 1412), ('bge_candidate859', 1415), ('bge_candidate1071', 1418), ('bge_candidate1072', 1419), ('bge_candidate1109', 1420), ('bge_candidate1146', 1421), ('bge_candidate1187', 1422), ('bge_candidate21', 1423), ('bge_candidate43', 1424), ('bge_candidate1', 1425)]

Cluster 212 (size: 2)

[('bge_candidate1124', 641), ('bge_candidate155', 642)]

Cluster 213 (size: 2)

[('bge_candidate548', 660), ('bge_candidate149', 661)]

Cluster 216 (size: 2)

[('bge_candidate1104', 693), ('bge_candidate131', 694)]

Cluster 217 (size: 2)

[('bge_candidate508', 713), ('bge_candidate124', 714)]

Cluster 219 (size: 2)

[('bge_candidate390', 718), ('bge_candidate121', 719)]

Cluster 222 (size: 2)

[('bge_candidate1009', 746), ('bge_candidate112', 747)]

Cluster 225 (size: 5)

[('bge_candidate1196', 780), ('bge_candidate136', 781), ('bge_candidate161', 782), ('bge_candidate1161', 783), ('bge_candidate100', 784)]

Cluster 226 (size: 2)

[('bge_candidate1156', 789), ('bge_candidate98', 790)]

Cluster 229 (size: 6)

[('bge_candidate410', 832), ('bge_candidate911', 833), ('bge_candidate102', 834), ('bge_candidate1349', 835), ('bge_candidate630', 836), ('bge_candidate81', 837)]

Cluster 231 (size: 6)

[('bge_candidate949', 938), ('bge_candidate1091', 939), ('bge_candidate1261', 940), ('bge_candidate1407', 941), ('bge_candidate626', 942), ('bge_candidate52', 943)]

Cluster 232 (size: 3)

[('bge_candidate200', 944), ('bge_candidate1075', 945), ('bge_candidate51', 946)]

Cluster 234 (size: 2)

[('bge_candidate1225', 966), ('bge_candidate44', 967)]

Cluster 235 (size: 8)

[('bge_candidate586', 1097), ('bge_candidate660', 1098), ('bge_candidate1086', 1099), ('bge_candidate1194', 1100), ('bge_candidate284', 1101), ('bge_candidate692', 1102), ('bge_candidate899', 1103), ('bge_candidate19', 1104)]

References

1. Wittkop T, Emig D, Lange S, Rahmann S, Albrecht M, et al. (2010) Partitioning biological data with transitivity clustering. Nat Methods 7: 419-420.

2. Cline MS, Smoot M, Cerami E, Kuchinsky A, Landys N, et al. (2007) Integration of biological networks and gene expression data using Cytoscape. Nat Protoc 2: 2366-2382.

3. Newman MEJ (2006) Finding community structure in networks using the eigenvectors of matrices. Phys Rev E 74: 036104.
