# Supplementary material for: Deep Sequencing of Organ- and Stage-Specific microRNAs in the Evolutionarily Basal Insect Blattella germanica (L.) (Dictyoptera, Blattellidae)
Source: PLoS One. 2011 Apr 28;6(4):e19350. doi: 10.1371/journal.pone.0019350 (PMC3084283; doi:10.1371/journal.pone.0019350)

**Text S2. Identification of miRNA precursors in *B. germanica* RNA databases**

We also looked for all putative miRNA candidates in a database of assembled contigs of *B. germanica* constructed by the integration of the two small RNA libraries approached in the current study (WB-6 and Ov-A), and other five RNA libraries of whole-body and ovary from cockroaches in different developmental stages (X. Bellés and M. D. Piulachs, unpublished RNA libraries from *B. germanica* ). As the cockroach *B. germanica* has not genome sequenced to date we constructed a *de novo* assembly pipeline (Figure 2A) which uses Velvet [1], SOAPdenovo [2] and phrap (http://www.phrap.org) for assembly; To map the reads from the two libraries, WB-6 and Ov-A, into the contigs we used bwa [3] and SAMtools [4] and visualization of the mapping was done by Integrative genomics viewer [5].

All candidate sequences were aligned against *Blattella* RNA database in order to identify potential miRNA precursors among those contigs. Only contigs with at least two sequences located in different regions of the contig were further checked for folding stability using Mfold [6], and miRNA:miRNA* duplexes were predicted and validated by experimental analysis (PCR and sequencing) (see the main text). The pipeline used is presented in the Additional Figure S2.

Sequences of miRNA precursors (highlighted in green) and contigs validated in the present study:

>bge-miR-cand1

TGTGATGTGCATGTGGGCTTTCCGAAATTTAACAGGTGGCCCACAAGTACA

TCATTTC

>Contig3830 (bge-miR-cand239)

AGGTCGCGCCGGCTCGTACCCATATCCGCAGCAGGTCTCCAAGGTGAAAA

GCCTCTAGTCGATAGACTAATGTAGGTAAGGGAAGTCGGCAAATTGGATC

CGTAACTTCGGGATAAGGATTGGCTCTGAGGACCGGGGCGCGTCGGGCGT

GGTCGGGAAG

>Contig5887 (bge-miR-cand147)

CGTCCGCGGCCACCTCTGGAGGCGGCCCGGGGTGGTCCTGACTTAGGACT

ACCCCCGGTCGACTTCCCGGGTTTGGCCTGACTCGACCCCTGCTGGCGGG

GAG

>Contig7413 (bge-miR-cand737)

TAAGCGTTTGCACCGTTGCCATTACGATCGCGGAAAAGTCCGCGCTCGAA

ACCCTATGGGTAACTCCCGCACAGATAGCCTCGGCGGCACCGTGAATAGA

CACGATAGCCGCCAGAGGGATATCGCCGGAGGTACTCCAGGCACCTTGAT

CCAAGGGTCTGTCGCTGGCAGGCGAGGCATTTTCATGCTCATCCCCTGCT

GCTTCGGCACCCTCGGTCAGGTCTTGGCCAGATAGGAAATCCTGACTGTC

CATGATCTATTAAAGTC

>Contig7421 (bge-miR-cand959)

AACTATGACTCTCTTAAGGTAGCCAAATGCCTCGTCATCTAATTAGTGAC

GCGCATGAATGGATTAACGAGATTCCCACTGTCCCTATCTACTATCTAGC

GAAACCACTGCCAAGGGAACGGGCTTGGAGGAATCAGCGGGGAAAGAAGA

CCCTGTTGAGCTTGACTCTAGTCTGGCACTGTAAGGAGACATGAGAGGTG

TAGCATAAGTGGGAGGCGGGTTCTCCTTCGCGGGTCCCGTCGCCAGTGAA

ATACCACTACTTTCATCGTTTCTTTACTTACTCGGTTGGGCGGAGCGCGT

GCGTCGTATGCCACTCGCTGGCCCGGCGTCACGGTGTTCTCGCGCCAAGC

GCTTTCA

The following figures show the reads from the two libraries, WB-6 and Ov-A, mapped into the contigs found in the *B.germanica* RNA database. The top panel shows the contig size and sequence by color code (Green = Adenine; Orange = Guanine; Red = Thymine; Blue = Cytocine). Bottom panel shows the location and distribution of reads across the contig. Vertical bars indicate the number of counts of nucleotides in each position of the contig sequence. Horizontal bars show the location of reads and colored spots highlight the nucleotide variations. Reads from each library were mapped separately. The graphics were plotted by Integrative genomics viewer (James et al., 2011).


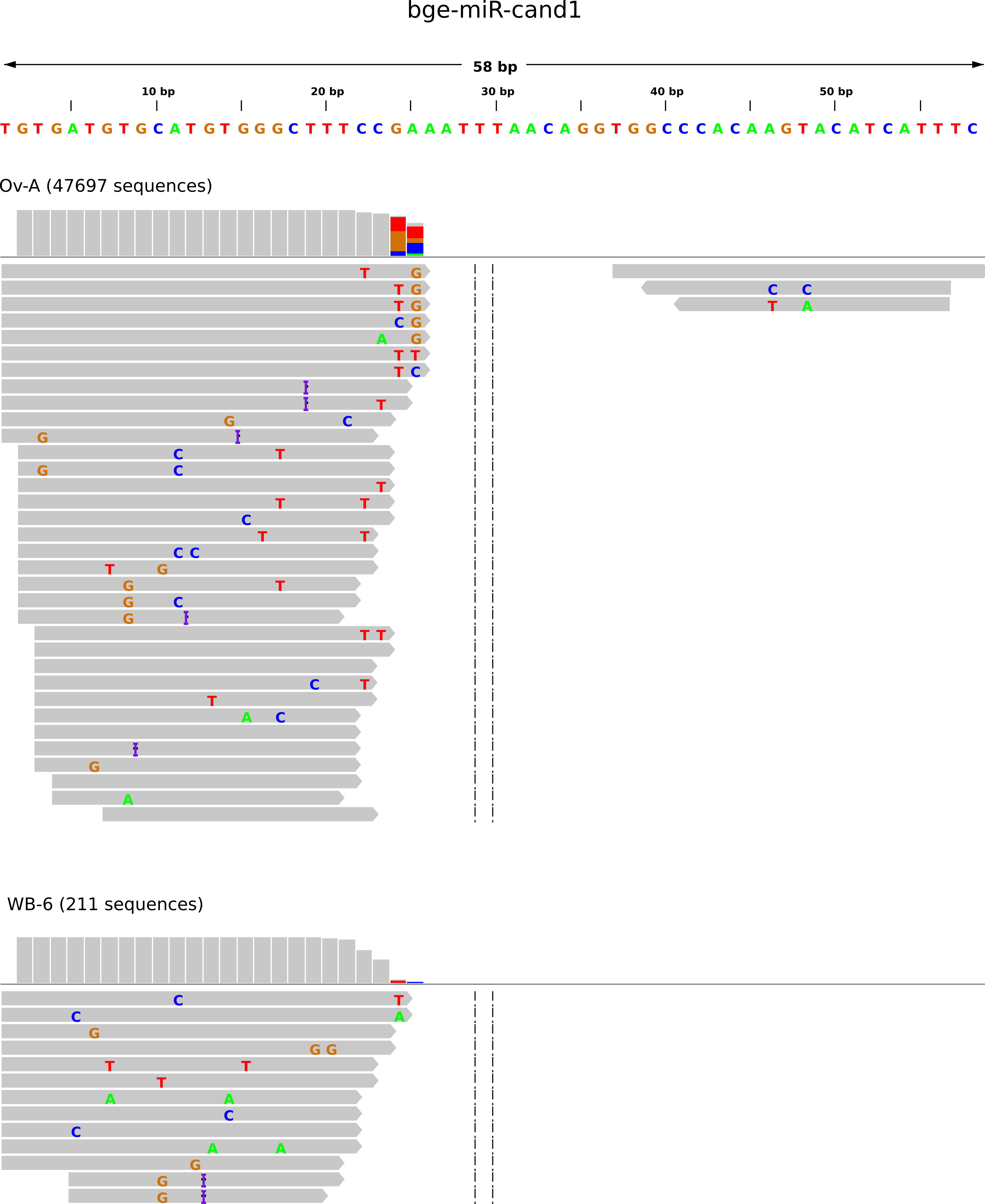


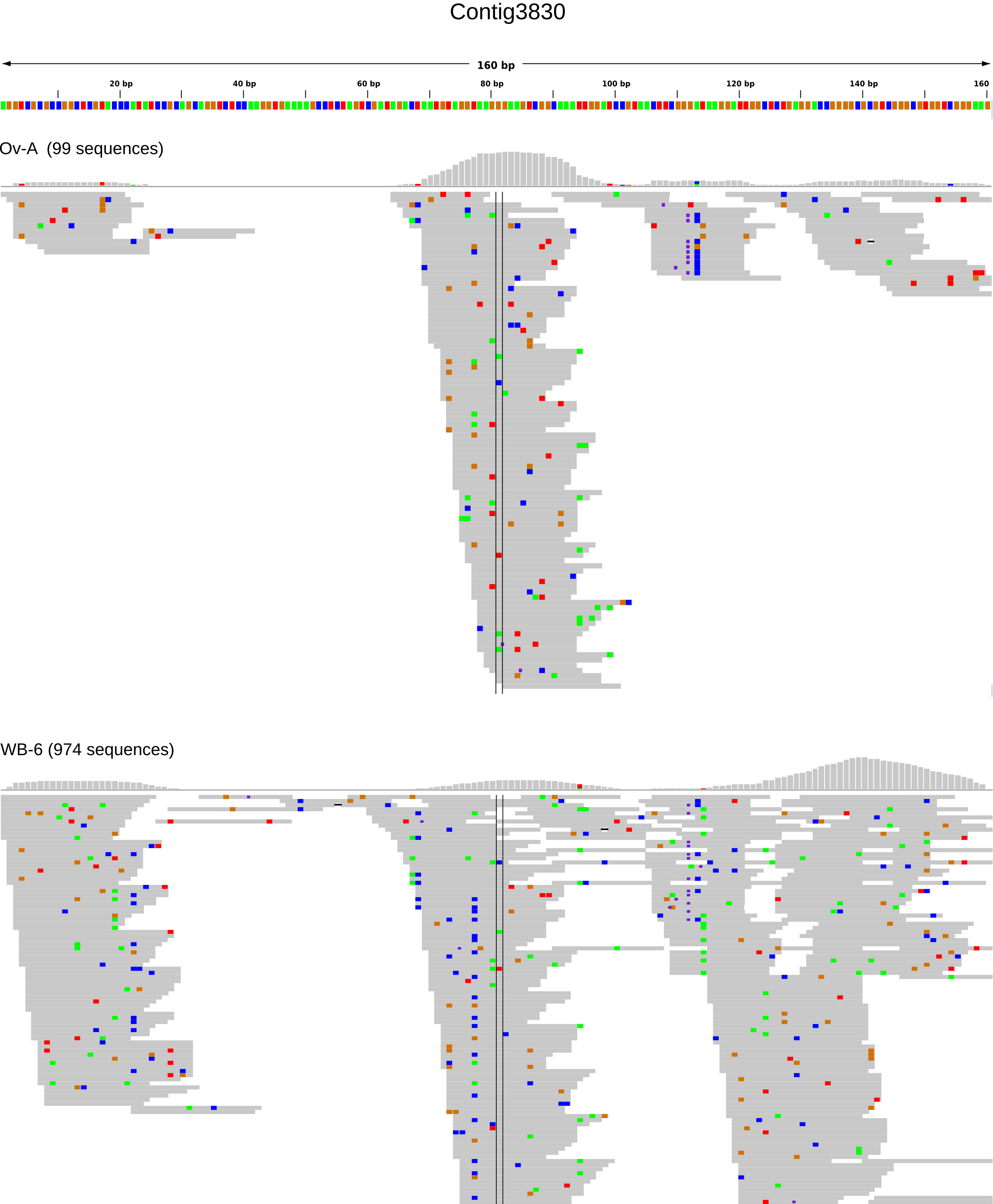


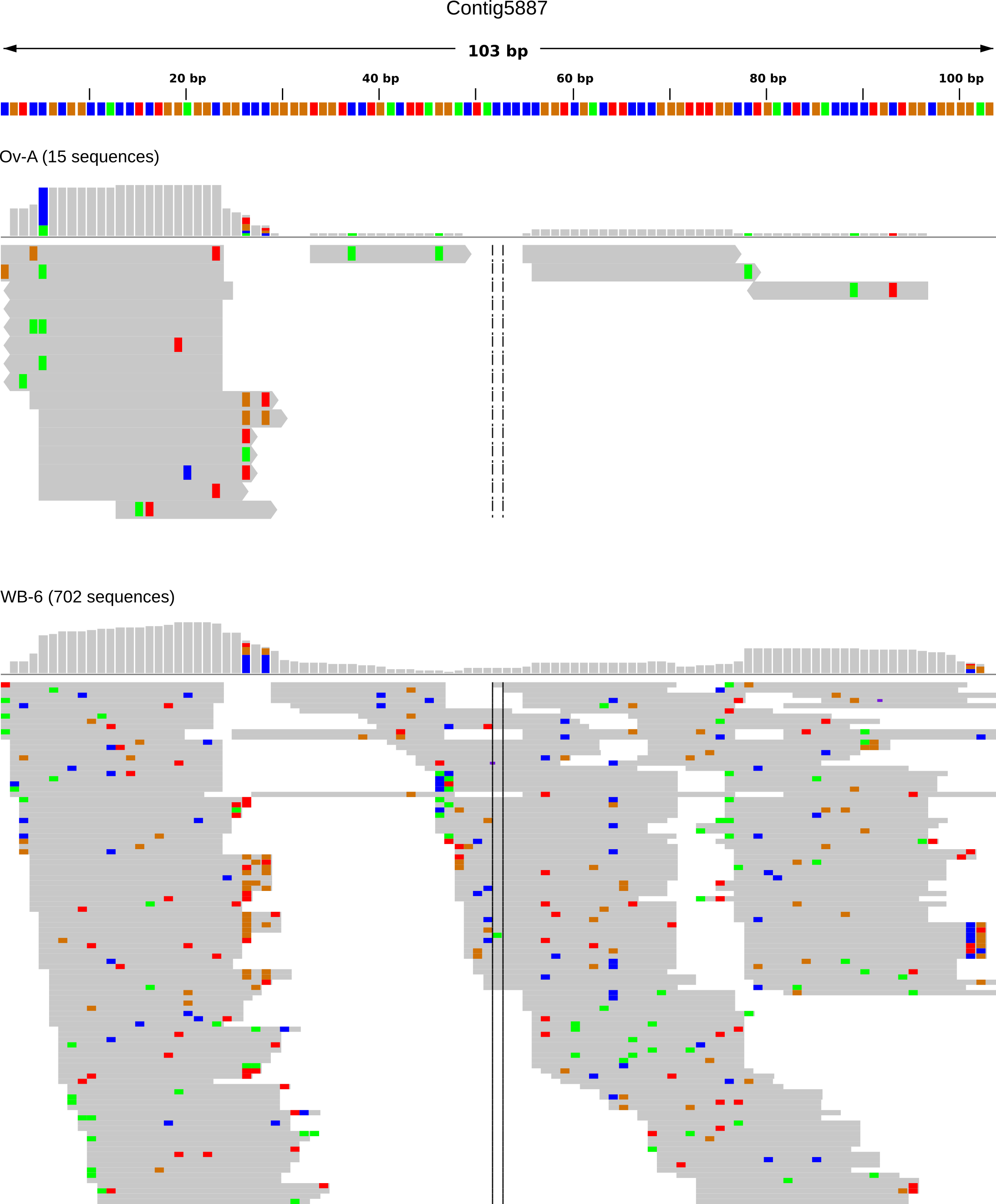


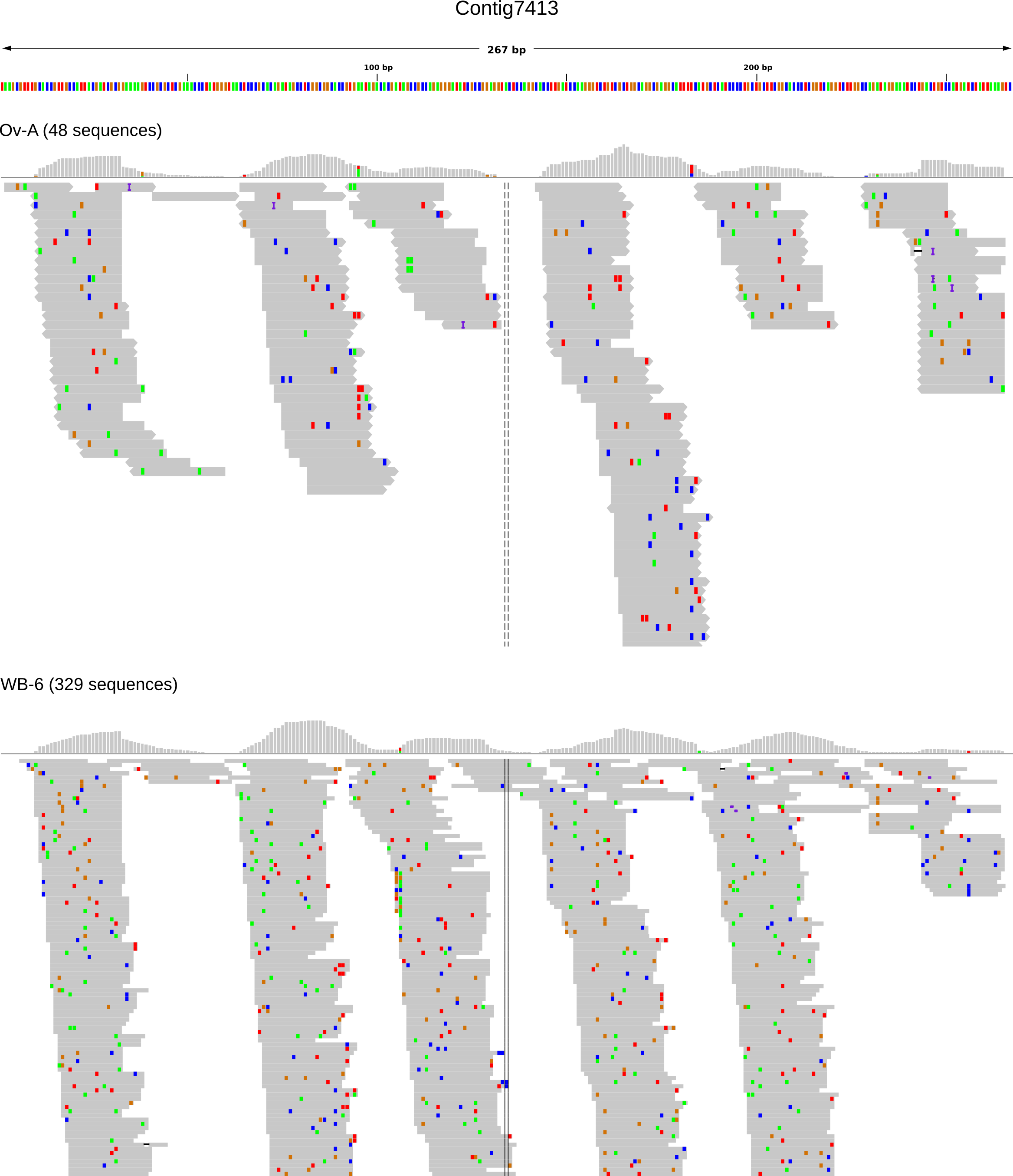


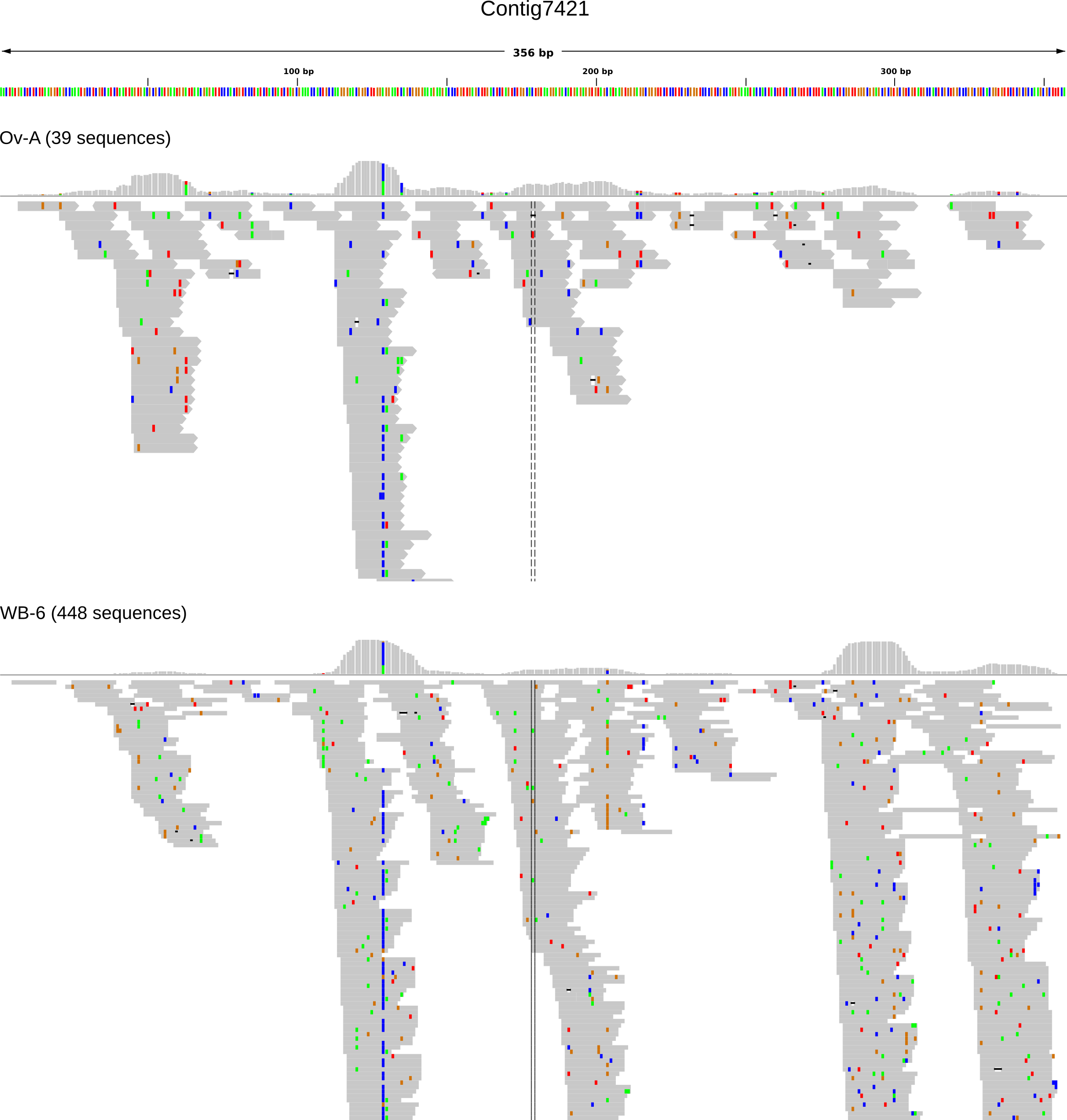

Supplement: Text S2 — Identification of miRNA precursors in B. germanica RNA databases. (DOC) [file pone.0019350.s013.doc]
